# Incremental Prognostic Value of Glucose Variability and the Lactate-to-Albumin Ratio Beyond APACHE II After a 48-Hour Landmark in Critically Ill Adults: A Retrospective Cohort Study

**DOI:** 10.3390/jcm15176925

**Published:** 2026-09-07

**Authors:** Sait Fatih Öner, Sevim Şenol Karataş, Oğuz Kağan Bulut

**Affiliations:** Department of Anesthesiology, Elazig Fethi Sekin City Hospital, University of Health Sciences, 23300 Elazig, Turkey; sfatihoner@gmail.com (S.F.Ö.); okaganbulut@hotmail.com (O.K.B.)

**Keywords:** intensive care, glucose variability, lactate-to-albumin ratio, mortality, prognostic model, APACHE II, landmark analysis

## Abstract

**Background/Objectives**: Glucose variability (GV) and the lactate-to-albumin ratio (LAR) have been associated with adverse outcomes in critically ill patients, but their incremental prognostic contribution beyond established severity assessment remains uncertain. This study evaluated the associations of GV and LAR with subsequent mortality and their incremental value beyond APACHE II in a conditional 48 h landmark cohort. **Methods**: This single-center retrospective cohort study included 384 critically ill adults who were alive and remained in the ICU through 48 h and had sufficient glucose, lactate, and albumin measurements. GV was quantified using the coefficient of variation (CV) from six glucose measurements closest to 0, 8, 16, 24, 32, and 48 h. The primary outcome was all-cause mortality during the 7 days following the 48 h landmark. Firth penalized logistic regression was used for prognostic modeling. Incremental performance was assessed sequentially for APACHE II, APACHE II + LAR, and APACHE II + LAR + GV using discrimination, calibration, Brier score, bootstrap internal validation, and decision-curve analysis. Sensitivity analyses adjusted for mean glycemia and restricted predictor information to the first 24 h. **Results**: Eighty-eight patients (22.9%) died during the 7-day post-landmark period. APACHE II (OR: 1.19 per point, 95% CI: 1.12–1.27; *p* < 0.001), LAR (OR: 1.97 per unit, 95% CI: 1.48–2.69; *p* < 0.001), and GV (OR: 1.07 per 1-percentage-point increase in CV, 95% CI: 1.02–1.11; *p* = 0.002) were independently associated with mortality. The AUC increased from 0.804 for APACHE II alone to 0.844 after addition of LAR and to 0.857 after further addition of GV. The additional AUC increase attributable to GV after LAR was modest (ΔAUC = 0.013, 95% CI: −0.004 to 0.030; *p* = 0.145), although model fit and Brier performance improved. The optimism-corrected C-index of the integrated model was 0.851. GV remained independently associated with mortality after adjustment for mean glucose (OR: 1.06, 95% CI: 1.02–1.11; *p* = 0.002) and in the temporally matched 24 h analysis (OR: 1.05, 95% CI: 1.01–1.09; *p* = 0.008). **Conclusions**: Among critically ill adults who survived to a 48 h landmark, LAR and GV provided prognostic information beyond APACHE II. Most of the incremental improvement in discrimination was attributable to LAR, whereas GV provided a smaller additional contribution that remained consistent across sensitivity analyses. These findings support further evaluation of LAR and GV as complementary prognostic markers, but external validation is required before clinical implementation.

## 1. Introduction

Critically ill patients admitted to intensive care units (ICUs) have substantial short-term mortality risk because of severe physiological disturbance, organ dysfunction, and acute systemic illness. Reliable prognostic assessment is important for risk stratification in critically ill patients. Severity-of-illness and organ dysfunction scores, including the Acute Physiology and Chronic Health Evaluation II (APACHE II) and Sequential Organ Failure Assessment (SOFA), are widely used for this purpose [1,2,3]. However, such scores summarize selected clinical information over defined assessment periods and may not capture dynamic metabolic changes that develop during the early ICU course.

Glucose variability (GV) describes fluctuations in blood glucose over time rather than glycemic exposure at a single measurement. Critical illness is accompanied by neuroendocrine stress, systemic inflammation, insulin resistance, nutritional interventions, and glucose-lowering treatment, all of which may influence glucose trajectories [4,5]. A recent systematic review and meta-analysis including more than 160,000 critically ill patients found that greater GV, assessed using several variability metrics, including the coefficient of variation (CV), was consistently associated with increased short-term mortality [6]. Earlier observational work likewise reported a strong independent association between GV and mortality in a heterogeneous critically ill population [7]. However, these associations do not by themselves establish how much prognostic information GV adds to an established severity score, nor whether any incremental contribution is independent of mean glycemia.

The interpretation of glucose-based prognostic markers must also be considered in the context of glucose-management evidence in critical illness. The NICE-SUGAR trial demonstrated that intensive glucose control targeting near-normal glucose concentrations increased 90-day mortality and severe hypoglycemia compared with conventional glucose management [8]. A subsequent NICE-SUGAR analysis further showed an association between hypoglycemia and mortality while emphasizing that such observational associations do not establish causality [9]. These findings highlight that glucose patterns in critically ill patients reflect both disease processes and therapeutic interventions and reinforce the need to distinguish prognostic association from causal interpretation.

Lactate and albumin provide complementary clinical information. Lactate is commonly used as a marker of metabolic stress and impaired tissue perfusion in critical illness [10], whereas low albumin may reflect systemic inflammation, increased capillary permeability, nutritional status, and reduced physiological reserve [11,12]. The lactate-to-albumin ratio (LAR) combines these two routinely measured variables into a single composite marker [13]. Higher LAR has been associated with adverse outcomes in critically ill and septic populations [13,14], and a large nationwide ICU cohort reported meaningful prognostic performance for in-hospital mortality [15]. Nevertheless, whether the ratio offers predictive advantages over entering lactate and albumin separately remains uncertain.

A further methodological issue is the timing of prognostic assessment. GV requires serial observations and therefore cannot be calculated reliably at ICU admission. We used a 48 h landmark to allow sufficient longitudinal glucose information to characterize early variability. This interval represents a pragmatic balance: a 24 h window provides fewer serial measurements and a narrower period over which to characterize glycemic fluctuation, whereas a later landmark such as 72 h would further delay prognostic assessment and increase conditioning on prolonged ICU survival. The 48 h point should therefore be interpreted as an analytical assessment landmark rather than as a biological threshold. Importantly, a landmark design estimates prognosis only among patients who survive to and remain under observation at that time.

The novelty of the present study is therefore incremental rather than based on establishing new individual associations of GV or LAR with mortality. We examined whether LAR and GV provided additional prognostic information beyond APACHE II in critically ill adults who survived to a 48 h landmark, with all-cause mortality during the subsequent 7 days as the outcome. Incremental performance was evaluated sequentially as APACHE II alone, APACHE II + LAR, and APACHE II + LAR + GV. We additionally examined whether the contribution of GV persisted after adjustment for mean glucose and when all predictor information was restricted to the first 24 h, and whether LAR differed from modeling lactate and albumin separately. Model assessment included discrimination, calibration, bootstrap internal validation, and decision-curve analysis.

## 2. Materials and Methods

### 2.1. Study Design and Setting

This retrospective cohort study was conducted in the adult intensive care unit (ICU) of the Department of Anesthesiology and Reanimation at Elazığ Fethi Sekin City Hospital. Electronic medical records of consecutive adult patients admitted between 1 January 2023 and 31 December 2025 were reviewed retrospectively.

A 48 h landmark analysis was used because glucose variability required serial measurements collected over the early ICU course. The 48 h assessment window was chosen pragmatically to provide sufficient longitudinal glucose information to characterize early glycemic variability while avoiding a longer landmark, such as 72 h, that would further restrict the analysis to patients with prolonged ICU survival and delay prognostic assessment. A shorter 24 h window would provide fewer serial observations and a narrower interval over which to characterize glycemic fluctuations. Accordingly, the 48 h landmark should be regarded as an analytical assessment point rather than a biological threshold.

The target population for the present analysis comprised critically ill adults who were alive and remained in the ICU through the 48 h landmark and had sufficient glucose and laboratory measurements for calculation of the study variables. The primary estimand was the risk of all-cause mortality during the subsequent 7 days conditional on having reached this landmark. Thus, the study does not estimate mortality risk from the time of ICU admission and its findings are not applicable to patients who died before completion of the initial 48 h assessment period.

The study evaluated the prognostic associations of glucose variability (GV) and the lactate-to-albumin ratio (LAR) and their incremental contribution when added to APACHE II. Model performance was assessed using discrimination, calibration, internal validation, and decision-curve analysis. Reporting followed the Strengthening the Reporting of Observational Studies in Epidemiology (STROBE) statement [16].

### 2.2. Study Population and Patient Selection

Consecutive adult patients admitted to the ICU during the study period were assessed for eligibility using the electronic hospital information system and digital ICU records. Patients were eligible for the landmark cohort if they were aged 18 years or older, were alive and remained in the ICU at 48 h after admission, had at least six blood glucose measurements available during the first 48 h, and had both serum lactate and albumin measurements available within the first 24 h.

Patients who died before reaching the 48 h landmark were not eligible for the landmark analysis by design because the prognostic assessment required completion of the initial 48 h observation window. Additional exclusions were insufficient glucose measurements for calculation of GV, missing lactate or albumin measurements, incomplete or inconsistent clinical or laboratory records, and duplicate records. For patients with multiple ICU admissions during the study period, only the first eligible admission was analyzed.

**Figure 1:**
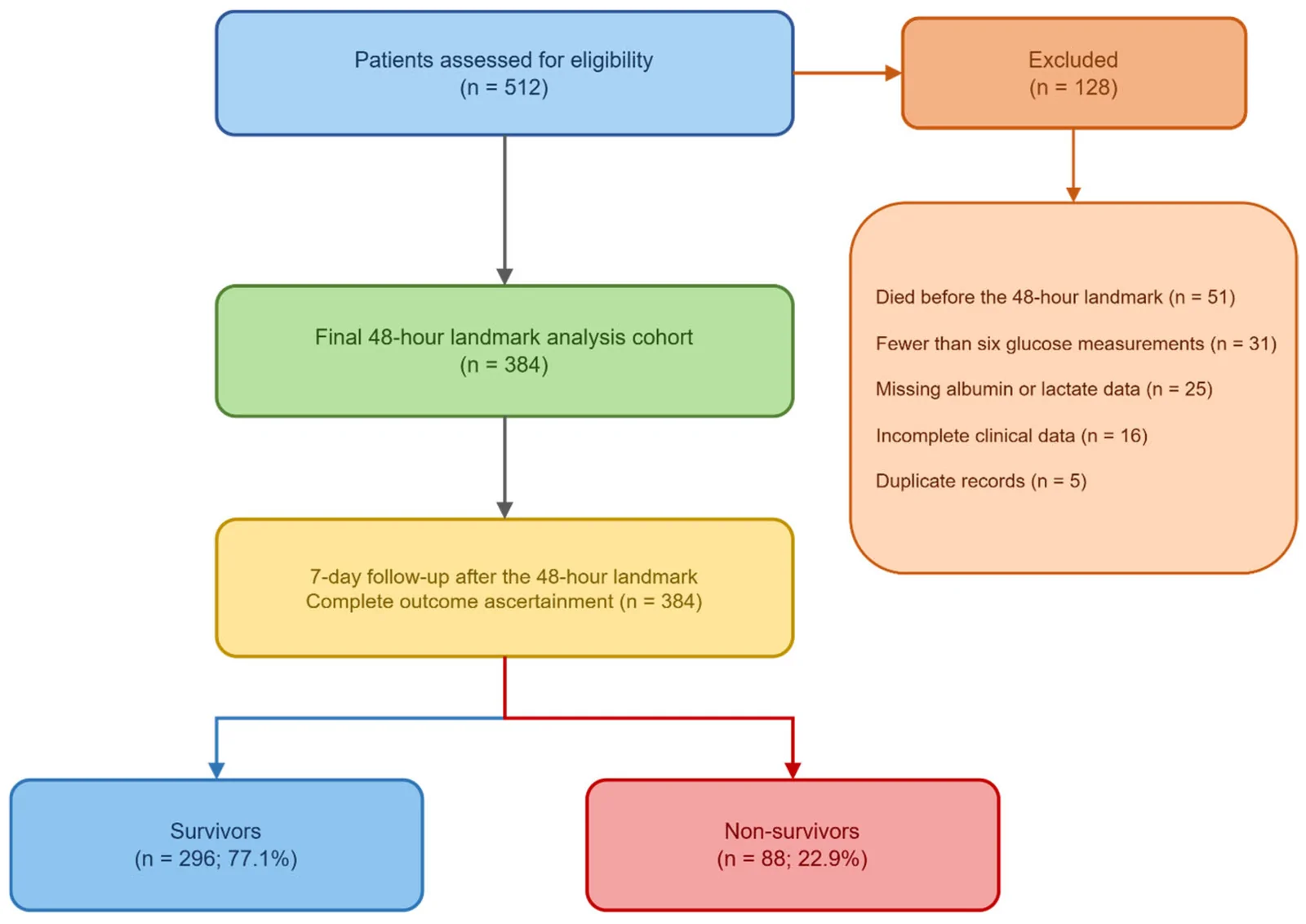
Flow chart of patient selection. Of 512 patients assessed for eligibility, 128 were excluded, including 51 who died before the 48 h landmark. The final landmark cohort comprised 384 patients who completed the initial 48 h assessment period and had complete predictor data; all had complete 7-day post-landmark outcome ascertainment.

The resulting analysis cohort therefore represented a conditional population of critically ill adults who survived and remained in the ICU through the 48 h landmark and had sufficient clinical and laboratory data for calculation of the study predictors. Patients with missing data required for the primary analysis were excluded before model development; no imputation was performed, and a complete-case approach was used. The patient-selection process is shown in Figure 1.

### 2.3. Data Collection and Variable Definitions

Patient demographic characteristics, primary ICU diagnosis, Glasgow Coma Scale (GCS) score at ICU admission, and APACHE II score calculated during the first 24 h after admission were obtained from the electronic hospital information system and digital ICU records. Serum lactate and albumin measurements obtained during the first 24 h and serial blood glucose measurements obtained during the initial 48 h assessment period were also extracted.

Blood glucose measurements were obtained from arterial or venous blood samples collected as part of routine clinical care. To provide a standardized temporal representation across patients, glucose variability (GV) was calculated using the glucose measurements closest to six target time points after ICU admission: 0, 8, 16, 24, 32, and 48 h. One measurement was retained for each target time point. During retrospective data abstraction, only the glucose values selected nearest the six target times were retained in the final analysis dataset; exact sampling timestamps, the complete glucose-testing history, and the total number of glucose measurements per patient were not retained. Consequently, deviations from target times, measurement frequency, and GV calculated from all available measurements could not be evaluated. The primary GV measure was the coefficient of variation (CV), calculated as

CV (%) = standard deviation of glucose/mean glucose × 100

CV was selected as the primary measure because it expresses glucose variability relative to the patient’s mean glucose concentration and therefore facilitates comparison of variability across patients with different average glycemic levels. Standard deviation was also calculated as an intermediate component of CV. Mean glucose during the 0–48 h period was calculated as the arithmetic mean of the same six glucose measurements and was used in sensitivity analyses to distinguish variability from overall glycemic exposure.

To examine whether the longer glucose assessment window influenced the incremental prognostic contribution of GV, a temporally matched 0–24 h sensitivity analysis was additionally performed. For this analysis, mean glucose and GV were recalculated using the four glucose measurements closest to 0, 8, 16, and 24 h. The 24 h GV measure was calculated using the same CV formula. Thus, APACHE II, LAR, mean glucose, and GV in this sensitivity analysis were all derived from information available within the first 24 h after ICU admission. The target population nevertheless remained the 48 h landmark cohort.

Mean amplitude of glycemic excursions (MAGE) was not calculated because reliable estimation of glycemic excursions requires a denser sequence of serial measurements than the six standardized target-time measurements available in the present retrospective dataset.

The lactate-to-albumin ratio (LAR) was calculated by dividing the first available lactate measurement by the corresponding albumin measurement within the first 24 h after ICU admission. Lactate and albumin were also retained as separate variables to allow sensitivity analyses comparing the composite LAR representation with models in which its two components were entered individually.

APACHE II scores were obtained from values automatically calculated by the hospital electronic system using the worst physiological measurements recorded during the first 24 h after ICU admission. GCS was recorded from the neurological assessment documented at ICU admission. Primary ICU diagnoses were categorized as sepsis, septic shock, pneumonia, trauma, postoperative care, or other causes according to the principal diagnosis recorded at admission. SOFA scores and sufficiently standardized organ-support variables were not systematically available for all patients and could not be reconstructed reliably; they were therefore not evaluated as alternative baseline models.

Information on insulin therapy, systemic corticosteroid administration, and nutritional support was available in the electronic records; however, reliable standardized information on treatment timing, dose, duration, and treatment changes across the 48 h assessment period was not available. These treatment variables were therefore not incorporated into the primary prognostic models. Because temporally resolved and quantitatively reliable treatment-exposure data were unavailable, treatment-adjusted sensitivity analyses were not performed.

### 2.4. Outcome Measures

The primary outcome was all-cause mortality during the 7 days following completion of the 48 h landmark assessment period. Mortality status was determined from the hospital information system and electronic ICU records.

All 384 patients included in the landmark cohort remained hospitalized throughout the 7-day post-landmark follow-up period. Therefore, outcome status was completely ascertainable for every included patient, with no loss to follow-up, post-discharge outcome uncertainty, or censoring during the outcome window.

Accordingly, the primary outcome represents conditional 7-day mortality among critically ill adults who survived to and completed the 48 h landmark assessment, rather than mortality calculated from the time of ICU admission. The prognostic models should therefore be interpreted as estimating subsequent short-term mortality risk at the 48 h landmark and not as admission-time prediction models.

### 2.5. Statistical Analysis

Statistical analyses were performed using R software (version 4.6.1; R Foundation for Statistical Computing, Vienna, Austria) in the Google Colaboratory environment [17]. All analyses were conducted in the conditional cohort of patients who reached the 48 h landmark. The dependent variable was all-cause mortality during the subsequent 7 days. All statistical tests were two-sided, and *p* < 0.05 was considered statistically significant.

Continuous variables were assessed using histograms, Q-Q plots, and the Shapiro–Wilk test. Normally distributed variables are presented as means ± standard deviations, whereas non-normally distributed variables are presented as medians (interquartile ranges). Categorical variables are reported as numbers and percentages. Survivor and non-survivor groups were compared using the independent-samples Student’s *t*-test or the Mann–Whitney U test for continuous variables and Pearson’s chi-square test or Fisher’s exact test for categorical variables, as appropriate.

No formal a priori sample-size calculation was performed because the study retrospectively included all consecutive patients who met the eligibility criteria during the predefined study period. The final landmark cohort comprised 384 patients, including 88 outcome events. The primary prognostic model contained three predictor terms, corresponding to approximately 29 outcome events per predictor. This event count is reported descriptively; no post hoc power calculation was performed.

The principal biomarkers of interest, GV and LAR, and APACHE II as the reference severity measure were defined on clinical and biological grounds and were consistent with the objectives of the original study protocol. Predictor inclusion in the primary model was not determined by a univariable *p*-value threshold. Univariable analyses were therefore used to describe individual associations rather than as a statistical screening procedure. Age and GCS were not entered separately into the primary model because these variables contribute to APACHE II, and lactate and albumin were not entered simultaneously with LAR in the primary model because they constitute the components of the ratio. The original ethics-approved study protocol is provided as Supplementary File S1, and clarifications regarding differences between the original protocol and the final analytical implementation are summarized in Supplementary Table S6.

Firth penalized logistic regression was used for univariable and multivariable prognostic analyses to reduce small-sample bias in regression coefficients. The primary integrated model included APACHE II score, LAR, and 48 h GV. Results are reported as odds ratios (ORs) with 95% profile-likelihood confidence intervals (CIs). GV was modeled continuously, with the OR expressed per 1-percentage-point increase in glucose CV.

Incremental prognostic performance was evaluated sequentially using three models: APACHE II alone; APACHE II plus LAR; and APACHE II plus LAR and GV. Discrimination was quantified using the area under the receiver operating characteristic curve (AUC), with 95% CIs. Paired changes in AUC between nested sequential models were assessed using DeLong’s method. Overall prediction error was assessed using the Brier score, and calibration was evaluated using the calibration intercept and calibration slope. As a secondary model-fit sensitivity analysis, nested conventional logistic-regression models were compared using likelihood-ratio tests.

To determine whether the prognostic contribution of GV was independent of overall glycemic exposure, mean glucose was calculated from the same six glucose measurements used for the 0–48 h GV calculation. Models containing APACHE II and mean glucose were compared with models additionally containing GV. A further sensitivity analysis evaluated GV after simultaneous adjustment for APACHE II, LAR, and mean glucose.

The temporal comparability of the predictor windows was examined in a separate sensitivity analysis restricted to information available during the first 24 h. Mean glucose and GV were recalculated from the measurements closest to 0, 8, 16, and 24 h. Models containing APACHE II, LAR, and 24 h mean glucose were compared with otherwise identical models additionally containing 24 h GV. This analysis used the same 48 h landmark cohort and was intended to assess whether the association of GV persisted when all predictor information was restricted to the first 24 h.

To examine whether LAR provided information distinct from simply entering its components separately, models containing APACHE II plus LAR were compared with models containing APACHE II plus lactate and albumin as separate terms. The corresponding models with GV added were also compared. These analyses were interpreted as comparisons of a parsimonious composite representation with separate component modeling rather than as tests of causal superiority of the ratio.

The functional form of GV was examined using restricted cubic spline (RCS) modeling with four knots located at the 5th, 35th, 65th, and 95th percentiles of the observed GV distribution. Overall GV association and departure from linearity were assessed using nested likelihood-ratio tests as sensitivity analyses. The primary Firth model containing a linear GV term was compared with an otherwise identical Firth model containing the RCS representation. Differences in apparent AUC were evaluated using 5000 paired bootstrap resamples. In addition to discrimination, Brier scores, calibration, and standardized predicted absolute mortality risks across the observed GV distribution were compared between the linear and RCS specifications. Individual differences in predicted probability were also examined to determine whether the small difference in global discrimination concealed clinically relevant differences in absolute-risk estimates.

Internal validation was performed using 1000 nonparametric bootstrap resamples with model refitting in each resample. Optimism was estimated as the difference between model performance in each bootstrap sample and performance of the bootstrap-fitted model when applied to the original cohort. Optimism-corrected C-index, Somers’ Dxy, Brier score, calibration intercept, and calibration slope were obtained by subtracting mean optimism from the corresponding apparent estimates. The sequential APACHE II, APACHE II + LAR, and APACHE II + LAR + GV models were validated separately. The mean-glycemia-adjusted and temporally matched 24 h sensitivity models were also subjected to bootstrap validation.

Decision-curve analysis was used to evaluate model net benefit over threshold probabilities ranging from 0.05 to 0.60. Net benefit was compared sequentially for APACHE II alone, APACHE II + LAR, and the integrated APACHE II + LAR + GV model, together with treat-all and treat-none strategies. Decision-curve analyses were also performed for the mean-glycemia-adjusted and temporally matched sensitivity models. Because threshold-specific net-benefit differences may vary across the probability range, these analyses were interpreted descriptively rather than as evidence of uniform clinical benefit.

Optimal cut-off values derived from ROC curves were determined using the Youden index. Because these thresholds were optimized within the development dataset, they were regarded as exploratory and not as externally validated clinical decision thresholds.

Patients missing variables required for the primary model were excluded before analysis, and no imputation was performed. Thus, the modeling analyses were complete-case analyses. No external or temporal validation cohort was used; all reported validation estimates therefore represent internal validation within the development cohort.

Analyses were implemented primarily using the logistf, pROC, dplyr, tibble, purrr, readxl, tidyr, ggplot2, and writexl R packages. The complete R analysis scripts and supporting reproducibility documentation, including a data dictionary and analytical workflow description, are publicly available in Zenodo (record 22142327) [18].

## 3. Results

### 3.1. Patient Selection and Cohort Characteristics

A total of 512 patients admitted to the adult ICU during the study period were assessed for eligibility. Of these, 51 died before reaching the 48 h landmark and were therefore not eligible for the landmark analysis. A further 77 patients were excluded because of fewer than six glucose measurements during the first 48 h (*n* = 31), missing albumin or lactate measurements (*n* = 25), incomplete clinical data (*n* = 16), or duplicate records (*n* = 5). The final 48 h landmark cohort therefore comprised 384 patients (Figure 1).

All 384 included patients had complete outcome ascertainment during the subsequent 7-day follow-up period. During this post-landmark interval, 296 patients (77.1%) survived and 88 (22.9%) died.

Baseline demographic, clinical, and laboratory characteristics are presented in Table 1. Non-survivors were older than survivors [70.9 (59.5–82.8) vs. 63.3 (53.8–72.8) years; *p* < 0.001], had higher APACHE II scores [23.0 (20.0–26.0) vs. 17.0 (12.8–20.0); *p* < 0.001], and had lower GCS scores [10.0 (8.0–11.0) vs. 12.0 (10.0–14.0); *p* < 0.001]. Serum albumin concentrations were lower among non-survivors [1.7 (1.4–2.1) vs. 2.2 (1.9–2.6) g/dL; *p* < 0.001], whereas serum lactate concentrations were higher [2.9 (2.2–4.3) vs. 1.7 (1.2–2.4) mmol/L; *p* < 0.001]. LAR [1.79 (1.20–2.69) vs. 0.80 (0.52–1.25); *p* < 0.001] and GV [15.5% (10.8–20.0) vs. 10.9% (6.8–16.0); *p* < 0.001] were also higher among non-survivors.

The proportion of male patients did not differ significantly between survivors and non-survivors (61.8% vs. 67.0%; *p* = 0.447). Diabetes mellitus was numerically more frequent among non-survivors, although the difference did not reach statistical significance (39.8% vs. 28.4%; *p* = 0.058). The distribution of primary ICU diagnoses differed between the groups (*p* < 0.001). Septic shock accounted for 9.5% of survivors and 29.5% of non-survivors; the corresponding proportions were 28.0% and 29.5% for sepsis, 28.4% and 23.9% for pneumonia, 10.8% and 6.8% for trauma, 9.8% and 4.5% for postoperative admission, and 13.5% and 5.7% for other diagnoses.

### 3.2. Univariable and Multivariable Prognostic Associations

In univariable Firth logistic regression analyses, age, APACHE II score, GCS score, serum albumin, serum lactate, LAR, and GV were each significantly associated with 7-day post-landmark mortality (Table 2). These univariable analyses were descriptive and were not used as a statistical screening procedure for selection of predictors into the primary multivariable model.

In the primary multivariable Firth penalized logistic regression model, APACHE II score, LAR, and GV each remained independently associated with mortality. Each 1-point increase in APACHE II score was associated with a 19% increase in the odds of mortality (OR: 1.19, 95% CI: 1.12–1.27; *p* < 0.001). Each 1-unit increase in LAR was associated with approximately twofold higher odds of mortality (OR: 1.97, 95% CI: 1.48–2.69; *p* < 0.001). Each 1-percentage-point increase in glucose CV was associated with a 7% increase in the odds of mortality (OR: 1.07, 95% CI: 1.02–1.11; *p* = 0.002).

### 3.3. Incremental Prognostic Performance of LAR and Glucose Variability

The APACHE II-only model showed an AUC of 0.804 (95% CI: 0.755–0.854) and a Brier score of 0.137. Addition of LAR increased the AUC to 0.844 (95% CI: 0.802–0.886), corresponding to an absolute AUC increase of 0.040 (95% CI: 0.014–0.065; *p* = 0.002 by paired DeLong test), while the Brier score decreased to 0.126. Sequential discrimination and internally validated performance of the three models are summarized in Table 3.

**Figure 2:**
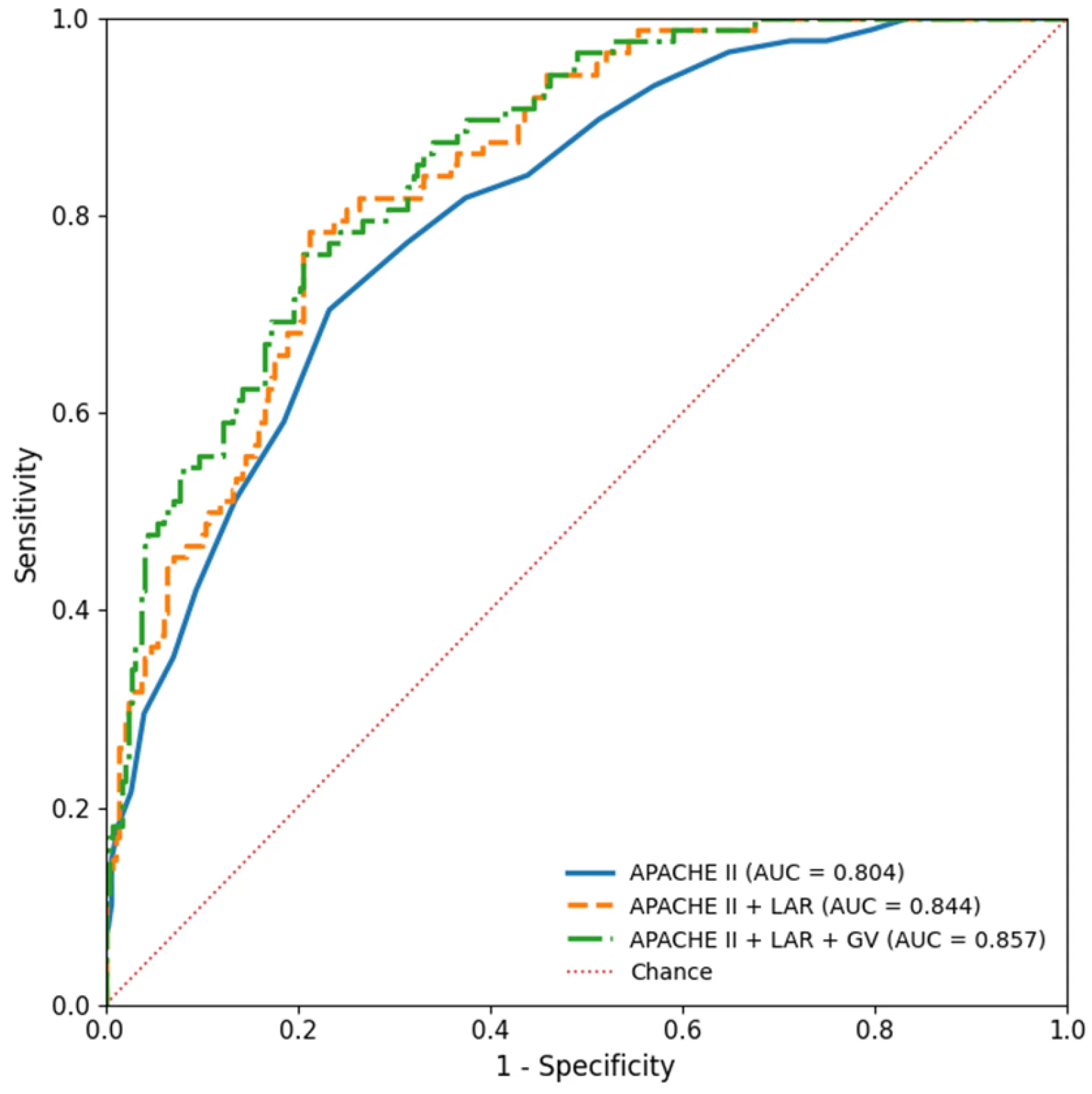
Receiver operating characteristic curves are shown for APACHE II alone, APACHE II + LAR, and the integrated APACHE II + LAR + GV model. The corresponding AUCs were 0.804 (95% CI: 0.755–0.854), 0.844 (95% CI: 0.802–0.886), and 0.857 (95% CI: 0.816–0.898), respectively. Addition of LAR to APACHE II increased the AUC by 0.040 (95% CI: 0.014–0.065; *p* = 0.002), whereas the subsequent addition of GV increased the AUC by 0.013 (95% CI: −0.004 to 0.030; *p* = 0.145).

Adding GV to the APACHE II + LAR model further increased the AUC to 0.857 (95% CI: 0.816–0.898) and reduced the Brier score to 0.121. The sequential ROC curves are shown in Figure 2. The incremental AUC increase attributable to GV after inclusion of LAR was 0.013 (95% CI: −0.004 to 0.030; *p* = 0.145). Despite the modest and statistically non-significant additional improvement in AUC, GV remained independently associated with mortality in the integrated model, and nested likelihood-ratio testing showed improved overall model fit after GV was added (likelihood-ratio χ^2^ = 9.93, df = 1, *p* = 0.002).

Compared with APACHE II alone, the fully integrated APACHE II + LAR + GV model improved the AUC by 0.053 (95% CI: 0.022–0.083; *p* < 0.001) and reduced the Brier score from 0.137 to 0.121. The larger discrimination increment was observed after addition of LAR, whereas GV provided a smaller additional contribution after LAR had already been incorporated.

### 3.4. Internal Validation and Calibration

Internal validation using 1000 bootstrap resamples showed limited optimism across the sequential models. The optimism-corrected C-index was 0.803 for APACHE II alone, 0.840 for APACHE II + LAR, and 0.851 for the integrated APACHE II + LAR + GV model. Corresponding optimism-corrected Brier scores were 0.138, 0.129, and 0.124, respectively. Thus, relative to APACHE II alone, the final integrated model improved the optimism-corrected C-index by 0.049 and reduced the optimism-corrected Brier score by 0.014. Adding GV after LAR yielded a smaller additional increase in the corrected C-index of 0.011 and a further reduction in the corrected Brier score of 0.005.

Calibration remained close to ideal after optimism correction. For the final integrated model, the optimism-corrected calibration intercept was −0.012 and the calibration slope was 0.985. The corresponding corrected Somers’ Dxy was 0.703. These findings indicate limited apparent overfitting within the development cohort, although they represent internal validation only and do not establish external transportability. Detailed apparent and optimism-corrected performance metrics for the sequential and LAR-adjusted sensitivity models are provided in Supplementary Table S2.

### 3.5. Mean Glycemia-Adjusted and Temporally Matched Sensitivity Analyses

In a mean-glycemia sensitivity analysis designed to distinguish glucose variability from overall glycemic exposure, the APACHE II + mean glucose model had an AUC of 0.811 (95% CI: 0.764–0.859) and a Brier score of 0.136. Addition of GV increased the AUC to 0.829 (95% CI: 0.783–0.875) and reduced the Brier score to 0.130. The corresponding ΔAUC was 0.018 (95% CI: −0.003 to 0.038; *p* = 0.092). In the extended model, GV remained independently associated with mortality (OR: 1.07 per 1-percentage-point increase in CV, 95% CI: 1.03–1.11; *p* < 0.001), whereas mean glucose was not independently associated with mortality (OR: 1.08 per 10 mg/dL increase, 95% CI: 0.97–1.20; *p* = 0.148).

In the 0–48 h mean-glycemia-adjusted sensitivity analysis, GV remained independently associated with 7-day post-landmark mortality after adjustment for APACHE II, LAR, and mean glucose (OR: 1.06 per 1-percentage-point increase in CV, 95% CI: 1.02–1.11; *p* = 0.002), whereas mean glucose was not independently associated with mortality (OR: 1.04 per 10 mg/dL increase, 95% CI: 0.93–1.16; *p* = 0.539). Addition of GV to the APACHE II + LAR + mean glucose model increased the AUC from 0.847 (95% CI: 0.806–0.888) to 0.857 (95% CI: 0.817–0.897). The corresponding ΔAUC was 0.010 (95% CI: −0.008 to 0.027; *p* = 0.275). Despite the modest change in discrimination, addition of GV improved overall model fit (likelihood-ratio χ^2^ = 9.85, df = 1, *p* = 0.002). After bootstrap correction, the C-index increased from 0.841 to 0.849 and the Brier score decreased from 0.131 to 0.126. The LAR-adjusted 0–48 h and temporally matched 0–24 h sensitivity analyses are summarized in Table 4.

A temporally matched sensitivity analysis was then performed using only predictor information available within the first 24 h, while retaining the same 48 h landmark cohort. In this analysis, 24 h GV remained independently associated with mortality after adjustment for APACHE II, LAR, and 24 h mean glucose (OR: 1.05 per 1-percentage-point increase in CV, 95% CI: 1.01–1.09; *p* = 0.008), whereas mean glucose was not independently associated with mortality (OR: 1.07 per 10 mg/dL increase, 95% CI: 0.96–1.20; *p* = 0.220). Addition of 24 h GV increased the AUC from 0.848 (95% CI: 0.807–0.889) to 0.852 (95% CI: 0.811–0.892), with a ΔAUC of 0.003 (95% CI: −0.012 to 0.019; *p* = 0.651). The nested likelihood-ratio test nevertheless indicated improved model fit (χ^2^ = 7.20, df = 1, *p* = 0.007). After bootstrap correction, the C-index changed from 0.841 to 0.843 and the Brier score from 0.131 to 0.127.

Taken together, these sensitivity analyses showed that the association of GV with subsequent mortality persisted after accounting for mean glycemia and when the predictor window was restricted to the first 24 h. However, the additional gain in discrimination was modest in both analyses. Key Firth regression coefficients from the mean-glycemia-adjusted and temporally matched sensitivity analyses are provided in Supplementary Table S1.

### 3.6. LAR Decomposition Sensitivity Analysis

To examine whether the composite LAR representation provided prognostic information beyond that obtained by modeling lactate and albumin separately, additional sensitivity analyses were performed. The APACHE II + LAR model yielded an AUC of 0.844 (95% CI: 0.802–0.886) and a Brier score of 0.126, whereas the model containing APACHE II with lactate and albumin entered as separate predictors yielded an AUC of 0.853 (95% CI: 0.812–0.893) and a Brier score of 0.123. The difference in AUC between these two specifications was small and not statistically significant (ΔAUC = 0.009, 95% CI: −0.004 to 0.021; *p* = 0.186). Comparisons of LAR with its individual components are also summarized in Table 4.

When GV was added, the APACHE II + LAR + GV model had an AUC of 0.857 (95% CI: 0.816–0.898) and a Brier score of 0.121, compared with an AUC of 0.865 (95% CI: 0.825–0.904) and a Brier score of 0.117 for the APACHE II + lactate + albumin + GV model. Again, the difference in discrimination was not statistically significant (ΔAUC = 0.007, 95% CI: −0.006 to 0.021; *p* = 0.272).

In the separate-component model, higher lactate remained associated with increased mortality (OR: 1.38, 95% CI: 1.14–1.68; *p* < 0.001), whereas higher albumin was associated with lower mortality (OR: 0.30, 95% CI: 0.16–0.57; *p* < 0.001). After GV was added, both lactate (OR: 1.35, 95% CI: 1.11–1.64; *p* = 0.002) and albumin (OR: 0.28, 95% CI: 0.14–0.54; *p* < 0.001) remained independently associated with mortality, and GV also remained independently associated with mortality (OR: 1.07, 95% CI: 1.03–1.12; *p* = 0.001). Corresponding coefficients from the LAR-component sensitivity models are also reported in Supplementary Table S1.

### 3.7. Nonlinearity and Restricted Cubic Spline Analysis

The functional form of GV was further examined using restricted cubic splines with knots at the 5th, 35th, 65th, and 95th percentiles of the observed GV distribution (3.58%, 9.39%, 15.2%, and 24.8%, respectively). In conventional nested logistic-regression sensitivity analyses, the overall GV contribution was significant (likelihood-ratio χ^2^ = 16.9, df = 3; *p* < 0.001), and there was evidence of departure from linearity (χ^2^ = 7.01, df = 2; *p* = 0.030).

The apparent AUC was 0.857 (95% CI: 0.816–0.898) for the linear GV model and 0.864 (95% CI: 0.824–0.904) for the RCS model. The absolute difference in AUC was 0.007, with a 5000-resample paired bootstrap 95% CI of −0.010 to 0.023 (*p* = 0.396). After 1000-resample bootstrap optimism correction, the C-index was 0.852 for the linear model and 0.854 for the RCS model; the corresponding corrected Brier scores were 0.124 and 0.123. Optimism-corrected calibration slopes were 0.990 and 0.970, respectively. Performance of the linear and RCS specifications is summarized in Table 4.

**Figure 3:**
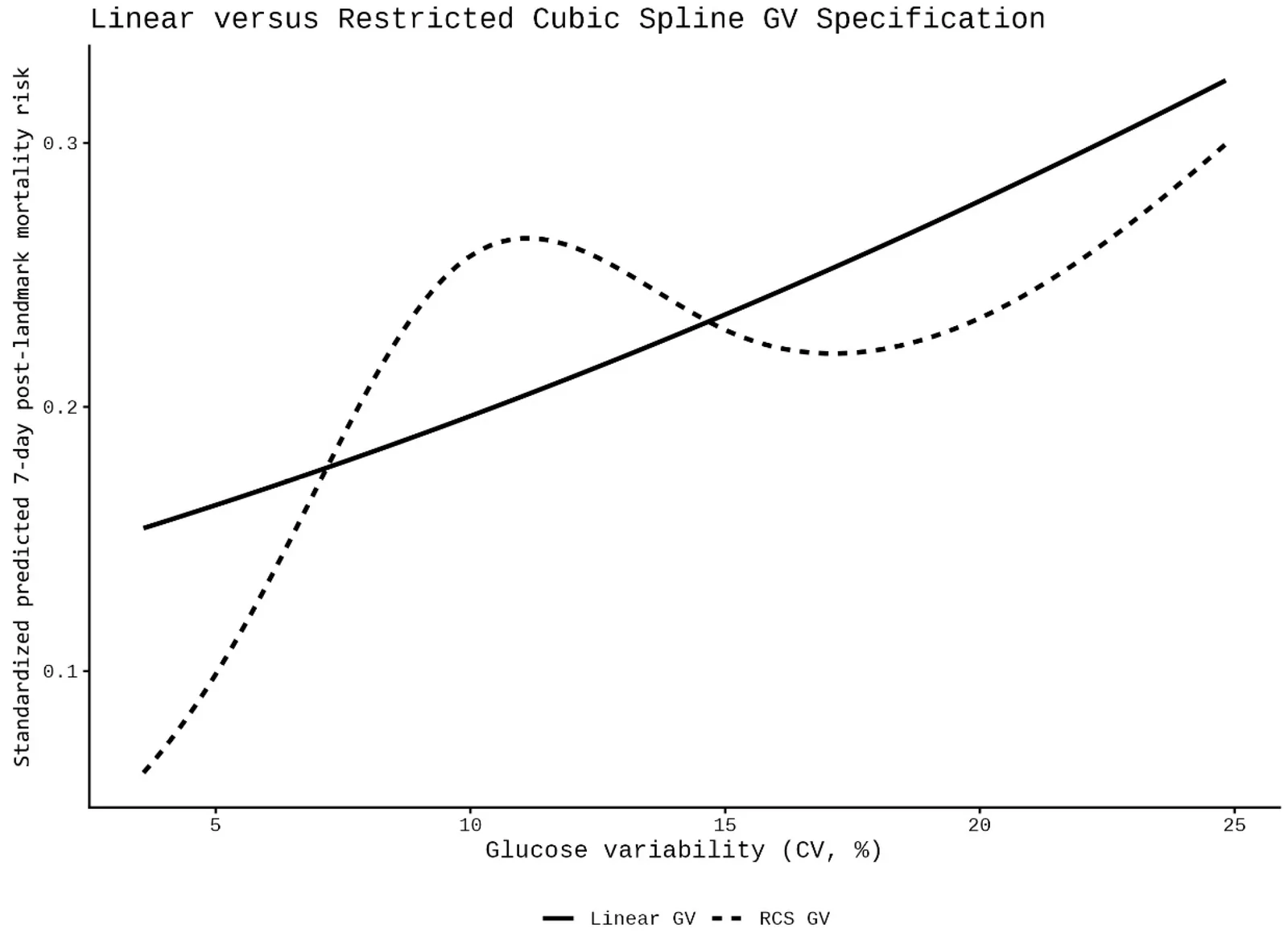
Standardized predicted 7-day post-landmark mortality risk according to glucose variability using linear and restricted cubic spline specifications. Standardized predicted mortality risks were estimated from models adjusted for APACHE II and the lactate-to-albumin ratio. The restricted cubic spline model used four knots located at the 5th, 35th, 65th, and 95th percentiles of the observed glucose-variability distribution (3.58%, 9.39%, 15.2%, and 24.8%, respectively). Evidence of nonlinearity was observed (likelihood-ratio χ^2^ = 7.01, df = 2; *p* = 0.030), although the difference in global discrimination between the linear and spline models was small (ΔAUC = 0.007, 95% bootstrap CI: −0.010 to 0.023; *p* = 0.396). APACHE II, Acute Physiology and Chronic Health Evaluation II; GV, glucose variability; CV, coefficient of variation; RCS, restricted cubic spline; AUC, area under the receiver operating characteristic curve; CI, confidence interval.

Despite the small difference in global discrimination, predicted absolute risks were not identical across the GV distribution. Standardized predicted mortality risks from the linear and RCS specifications are shown in Figure 3. At the 5th percentile of GV (3.58%), the standardized predicted mortality risk was 15.4% with the linear model and 6.2% with the RCS model; at the median GV value (11.5%), the corresponding risks were 20.8% and 26.3%; and at the 90th percentile (21.5%), they were 29.2% and 25.0%, respectively. Across all patients, the median absolute difference between individual linear and RCS predicted probabilities was 2.8 percentage points; 26.0% of patients had an absolute difference greater than 5 percentage points and 4.7% had a difference greater than 10 percentage points. Standardized mortality risks across selected GV percentiles and individual prediction differences between the linear and RCS specifications are detailed in Supplementary Table S4.

### 3.8. Decision-Curve Analysis

Decision-curve analysis was performed across threshold probabilities from 0.05 to 0.60. The integrated APACHE II + LAR + GV model showed greater net benefit than APACHE II alone throughout the evaluated threshold range. Addition of LAR to APACHE II accounted for a substantial proportion of this improvement. The corresponding sequential decision curves, including treat-all and treat-none strategies, are shown in Supplementary Figure S1.

The incremental net benefit of adding GV to the APACHE II + LAR model was smaller and varied according to the decision threshold. At lower and intermediate threshold probabilities, the difference between the two models was modest and was not uniformly positive. From approximately 0.30 upward, the integrated model generally showed greater net benefit than the APACHE II + LAR model, with the difference becoming more apparent at several higher thresholds. Accordingly, the decision-curve findings suggest greater net benefit of the integrated model than APACHE II alone within this development cohort but do not indicate uniform incremental benefit attributable to GV across all decision thresholds.

In the mean-glycemia-adjusted and temporally matched 24 h sensitivity analyses, the addition of GV likewise produced threshold-dependent changes in net benefit rather than consistent improvement across the full evaluated range. These sensitivity decision curves were therefore interpreted as supportive analyses rather than evidence of uniform clinical superiority. Threshold-specific net-benefit estimates for the primary and sensitivity decision-curve analyses are provided in Supplementary Table S3.

### 3.9. Exploratory ROC-Derived Cut-Offs

Exploratory cut-off values were derived within the development cohort by maximizing the Youden index. The optimal cut-off was 21.0 for APACHE II, with 70.5% sensitivity and 76.7% specificity; 1.32 for LAR, with 73.9% sensitivity and 78.7% specificity; and 9.75% for GV, with 85.2% sensitivity and 44.6% specificity. For the integrated APACHE II + LAR + GV model, the optimal predicted-probability threshold was 0.240, corresponding to 76.1% sensitivity and 79.4% specificity. The exploratory ROC-derived thresholds and corresponding sensitivity and specificity estimates are summarized in Supplementary Table S5.

Because these thresholds were optimized in the same cohort used for model development and were not externally validated, they should be interpreted as exploratory rather than as recommended clinical decision thresholds.

## 4. Discussion

### 4.1. Principal Findings

In this 48 h landmark cohort of critically ill adults, APACHE II, LAR, and glucose variability were independently associated with subsequent 7-day mortality. The principal incremental gain in discrimination occurred when LAR was added to APACHE II, whereas GV provided a smaller additional contribution. Specifically, the AUC increased from 0.804 with APACHE II alone to 0.844 after addition of LAR and to 0.857 after further addition of GV. Although the additional increase in AUC attributable to GV after LAR was modest and not statistically significant, GV remained independently associated with mortality and improved overall model fit and prediction error, as reflected by the likelihood-ratio test and Brier score.

The association of GV was consistent across several sensitivity analyses. GV remained independently associated with mortality after adjustment for mean glycemia and when the predictor window was restricted to the first 24 h, although the corresponding gains in discrimination remained small. Internal bootstrap validation showed limited optimism, with an optimism-corrected C-index of 0.851 for the integrated model and a calibration slope close to unity. However, the additional net benefit attributable specifically to GV was threshold-dependent rather than uniform across the decision-curve range.

Additional analyses also refined the interpretation of LAR and the functional form of GV. Modeling lactate and albumin as separate predictors produced numerically similar or slightly higher discrimination than using LAR, with no statistically significant difference between the two approaches. Thus, the present findings support LAR primarily as a parsimonious composite representation rather than as a superior alternative to its individual components. Restricted cubic spline analysis demonstrated evidence of nonlinearity in the association between GV and mortality. Although the spline model improved global discrimination only minimally, predicted absolute risks differed meaningfully from those of the linear model in some regions of the GV distribution, indicating that a simple linear specification may not fully capture individual-level risk relationships.

### 4.2. Glucose Variability, Mean Glycemia, and Temporal Comparability

Previous observational evidence has consistently linked greater GV with adverse outcomes in critically ill patients. Krinsley et al. reported a strong independent association between glycemic variability and mortality in a heterogeneous critically ill population [7], and a recent systematic review and meta-analysis similarly found that greater GV was associated with increased short-term mortality across several variability metrics [6]. More recently, a multicenter prospective observational study showed that glucose CV measured during the first 24 h was independently associated with 28-day mortality [19]. Our findings extend this literature by examining GV as an incremental prognostic marker within an explicitly defined 48 h landmark framework.

An important consideration is that CV is mathematically dependent on mean glucose and therefore cannot be interpreted independently of overall glycemic exposure without additional analysis. In the present study, 48 h GV remained associated with subsequent mortality after simultaneous adjustment for APACHE II, LAR, and mean glucose, whereas mean glucose itself was not independently associated with mortality in that model. Moreover, when GV and mean glucose were recalculated using only measurements available during the first 24 h, GV remained independently associated with mortality. The latter analysis reduced the temporal mismatch between APACHE II/LAR and GV, although it did not remove the selection inherent in conditioning the analysis on survival to the 48 h landmark. Importantly, the additional gains in AUC were modest in both sensitivity analyses, indicating that the consistency of the GV association across sensitivity analyses should not be equated with a large incremental improvement in discrimination.

The interpretation of glycemic patterns in critical illness should also be considered in the context of glucose-management trials. The NICE-SUGAR trial demonstrated that intensive glucose control targeting near-normal concentrations increased 90-day mortality and markedly increased severe hypoglycemia compared with conventional glucose control [8]. A subsequent NICE-SUGAR analysis found that both moderate and severe hypoglycemia were associated with increased risk of death, while explicitly noting that the observational nature of this secondary association could not establish causality [9]. These findings underscore that glucose trajectories in the ICU reflect not only underlying illness but also therapeutic interventions and their consequences. Accordingly, GV in our study should be interpreted as a prognostic marker derived from the observed clinical course rather than as evidence that glycemic fluctuation itself causes mortality.

Several biological mechanisms have nevertheless been proposed to explain why fluctuating glucose concentrations might accompany adverse outcomes. The experimental and mechanistic literature has linked intermittent hyperglycemic exposure with oxidative stress, endothelial dysfunction, inflammatory activation, altered endothelial protective pathways, and cellular injury [20,21,22,23]. These mechanisms provide biological plausibility for an association between GV and adverse outcomes, but they do not establish that reducing GV would improve survival in critically ill patients. In this retrospective cohort, GV may also reflect disease severity, nutritional exposure, insulin administration, corticosteroid treatment, or other aspects of ICU management. The present results therefore support prognostic association rather than a causal biological effect.

### 4.3. Lactate-to-Albumin Ratio and Its Individual Components

Lactate and albumin capture different but potentially complementary aspects of critical illness. Elevated lactate may accompany impaired tissue perfusion and reduced lactate clearance [10,24], whereas low albumin may reflect systemic inflammation, increased capillary permeability, nutritional status, and reduced physiological reserve [11,12]. Combining these variables as the lactate-to-albumin ratio has therefore been proposed as a pragmatic marker integrating acute metabolic stress with underlying biological reserve.

Previous studies have associated higher LAR with mortality in critically ill populations, particularly among patients with sepsis and septic shock [13,24,25,26]. Systematic reviews and meta-analyses have likewise supported an association between elevated LAR and adverse outcomes in sepsis [14,26], while a recent nationwide ICU cohort also reported the prognostic utility of LAR across a broader intensive-care population [15]. Our findings are consistent with this literature in showing a strong independent association between LAR and subsequent mortality and a clear improvement in discrimination when LAR was added to APACHE II.

However, the present sensitivity analysis provides an important qualification. Modeling lactate and albumin as separate predictors produced numerically slightly higher discrimination than modeling LAR, although the differences were small and not statistically significant. This finding does not support the interpretation that LAR is intrinsically superior to its individual components. Rather, LAR appears to offer a parsimonious one-term composite representation of two strongly prognostic variables. The ratio may be attractive for bedside interpretation and model simplicity, but its mathematical construction can also obscure the distinct contributions of lactate and albumin. Accordingly, the present results support LAR as a compact prognostic representation rather than as evidence that the ratio contains unique biological information unavailable from its components.

Another limitation of LAR in the present study is that it was derived from a single lactate and albumin assessment within the first 24 h. Serial lactate measurements and lactate clearance may provide additional information regarding the evolution of tissue perfusion and response to treatment. Shadvar et al. directly compared LAR with lactate and lactate clearance in septic shock and demonstrated that these related markers can provide overlapping but not identical prognostic information [24]. Because serial lactate trajectories and standardized clearance measurements were not available in the present dataset, we could not determine whether dynamic lactate indices would outperform or complement the single-time LAR measure. Future studies should compare LAR with serial lactate, lactate clearance, and repeated albumin measurements within the same prognostic framework.

Finally, most previous LAR evidence has been generated in sepsis or septic shock cohorts [14,24,25,26]. The present cohort included a broader spectrum of ICU diagnoses, suggesting that the association may extend beyond infection-related critical illness. Nevertheless, this broader applicability should be confirmed in external multicenter cohorts before LAR is incorporated into routine risk-prediction strategies.

### 4.4. Incremental Value Beyond APACHE II and Clinical Interpretation

APACHE II remains an established benchmark for severity-of-illness assessment in critically ill patients, while organ dysfunction scores such as SOFA provide complementary approaches to clinical risk stratification [2,3]. The purpose of the present model was therefore not to replace established severity scoring, but to determine whether routinely available metabolic information collected during the early ICU course could add prognostic information to APACHE II at a defined 48 h assessment point.

The sequential analyses clarify the relative contribution of the two added biomarkers. Most of the improvement in discrimination beyond APACHE II occurred after addition of LAR, whereas GV produced a smaller additional increment. Nevertheless, the contribution of GV was supported by its independent regression association, improvement in overall model fit and Brier score, and preservation of these findings after adjustment for mean glycemia and restriction of the predictor window to the first 24 h. Thus, the principal contribution of GV in this dataset is better characterized as additional prognostic information rather than a large increase in discrimination.

Internal validation was reassuring, with limited optimism and calibration slopes close to unity after bootstrap correction. Decision-curve analysis also showed greater net benefit for the integrated model than for APACHE II alone across the evaluated threshold range. However, the incremental net benefit specifically attributable to GV after LAR had been added was smaller and threshold-dependent. These findings support further evaluation of the integrated model but do not establish that use of the model would improve patient outcomes or clinical decision-making.

The clinical interpretation of the model is also constrained by its landmark design. Prediction is made after completion of the initial 48 h observation period and applies only to patients who have survived to that point and have sufficient measurements to calculate the predictors. It should therefore not be interpreted as an admission-time mortality model or applied to patients who die during the first 48 h. This distinction is particularly important when comparing its performance with APACHE II, which is derived from information obtained during the first 24 h.

Finally, the present model should be regarded as an internally validated development-stage prognostic model rather than a ready-to-use bedside calculator. The ROC-derived thresholds were optimized within the same cohort and should be considered exploratory. External validation in independent ICUs, ideally including contemporary severity measures and organ-support variables, is required to assess calibration, transportability, and clinical utility before any implementation can be considered.

### 4.5. Strengths and Limitations

This study has several strengths. First, it addressed a clearly defined conditional prediction question using a 48 h landmark framework and explicitly separated the predictor-assessment period from the subsequent outcome window. Second, the incremental contributions of LAR and GV were examined sequentially rather than evaluating only the final integrated model. Third, several additional sensitivity analyses were used to examine the consistency of the findings, including adjustment for mean glycemia, restriction of all predictor information to the first 24 h, comparison of LAR with lactate and albumin entered separately, and assessment of nonlinear GV effects. Finally, model performance was evaluated beyond apparent AUC by incorporating calibration, Brier score, bootstrap optimism correction, and decision-curve analysis. The use of internal validation and calibration assessment is consistent with contemporary recommendations for prognostic-model evaluation [27]. Complete 7-day post-landmark outcome ascertainment was available for all patients in the analysis cohort.

Several limitations require careful consideration. First, this was a single-center retrospective study, and both the case mix and local ICU management practices may differ from those in other institutions. Residual confounding from unmeasured clinical characteristics and changes in treatment practice cannot be excluded. The findings should therefore be regarded as development-stage prognostic evidence rather than proof of generalizability or clinical effectiveness.

Second, the landmark design necessarily created a conditional survivor cohort. Fifty-one screened patients died before reaching the 48 h landmark and were therefore not eligible for the prediction question addressed by the study. An additional 77 patients were excluded because of insufficient glucose measurements, missing lactate or albumin data, incomplete clinical data, or duplicate records. Patient-level covariate data sufficient for a valid comparison between the included cohort and all excluded patients were not available. Consequently, the degree and direction of selection bias introduced by landmark survival and biomarker availability could not be quantified. The complete-case approach may introduce additional selection because the availability of glucose, lactate, and albumin measurements in routine ICU practice is unlikely to be completely random. These limitations restrict the applicability of the model to patients who survive to 48 h and have sufficient measurements for predictor calculation.

Third, glucose measurements were obtained during routine clinical care rather than according to a prospectively standardized sampling protocol. Although measurements closest to the six target time points were selected to create a common temporal structure across patients, the exact deviations between actual sampling times and target times and the total number of glucose measurements obtained for each patient were not available in the analysis dataset. Clinically unstable patients may have undergone more frequent glucose testing, and GV could therefore partly reflect monitoring intensity in addition to underlying glycemic physiology. A sensitivity analysis based on all available glucose measurements or adjustment for measurement frequency could not be performed. This represents a potential source of informative-sampling bias.

Fourth, GV is influenced by both disease processes and treatment. Insulin administration, corticosteroids, nutritional support, dextrose exposure, vasopressor therapy, renal replacement therapy, and other aspects of ICU management may alter glucose trajectories while also being related to prognosis. Although information on some treatment exposures was present in the electronic records, reliable standardized data on their timing, dose, duration, and changes during the glucose-assessment period were not available. Incorporating simple ever/never treatment indicators without temporal information could itself introduce substantial exposure misclassification. Treatment-adjusted sensitivity analyses were therefore not performed. GV should consequently be interpreted as a prognostic marker measured within an observational treatment environment rather than as an independent causal exposure.

Fifth, APACHE II was used as the reference severity measure because it was systematically available in the study dataset. SOFA scores and sufficiently standardized organ-support variables were not available for all patients and could not be reconstructed reliably retrospectively. The study therefore cannot determine whether GV and LAR provide similar incremental information beyond alternative contemporary severity assessments or detailed measures of respiratory, circulatory, and renal support.

Sixth, GV was characterized primarily using CV calculated from six target-time measurements. Although CV permits comparison of variability relative to mean glycemia, it does not capture all aspects of glucose dynamics. Metrics such as MAGE, MODD, time in range, hypoglycemic exposure, or continuous-glucose-monitoring-derived variability could not be evaluated. In particular, MAGE requires a sufficiently dense sequence of glucose measurements to identify clinically meaningful excursions and could not be estimated reliably from the available six-point retrospective sampling structure. The 24 h matched-window analysis and adjustment for mean glucose strengthened interpretation of the CV findings but do not eliminate this measurement limitation.

Seventh, LAR was calculated from lactate and albumin measurements obtained within the first 24 h and did not incorporate serial lactate trajectories or lactate clearance. Dynamic changes in lactate may provide additional prognostic information and should be compared directly with LAR in future prospective studies.

Eighth, although the RCS analysis identified evidence of nonlinearity, the linear GV specification was retained as the primary parsimonious model. The difference in overall discrimination between the linear and spline models was small after bootstrap correction; however, individual predicted probabilities differed appreciably in a subset of patients. Therefore, the linear model should not be interpreted as providing fully interchangeable individual risk estimates across the entire GV distribution, particularly without external validation.

Finally, validation was limited to bootstrap resampling within the development cohort. Although the low estimated optimism and near-unity calibration slopes are reassuring regarding internal stability, they do not establish transportability to another ICU, time period, or glucose-monitoring environment. No independent external or temporal validation cohort was available. In addition, the outcome was limited to 7-day mortality after the 48 h landmark, so performance for in-hospital, 28-day, or longer-term mortality remains unknown. ROC-derived thresholds were optimized within the same development cohort and should therefore be regarded as exploratory rather than as clinical decision thresholds.

## 5. Conclusions

In critically ill adults who survived to a 48 h landmark, LAR and GV were independently associated with subsequent 7-day mortality beyond APACHE II. Most of the incremental improvement in discrimination was attributable to LAR, whereas GV provided a smaller additional prognostic contribution that persisted after adjustment for mean glycemia and in the temporally matched 24 h sensitivity analysis. The integrated model showed limited optimism after bootstrap internal validation, but the additional gain in discrimination and net benefit attributable specifically to GV was modest and threshold-dependent. These findings support further evaluation of LAR and GV as candidate complementary prognostic markers within a 48 h landmark framework. External validation in independent ICU cohorts is required before clinical implementation or use of the derived risk estimates and thresholds.

## Figures and Tables

**Table 1:** Baseline demographic, clinical, and laboratory characteristics of the 48 h landmark cohort according to 7-day post-landmark survival status.

Variable	Overall (*n* = 384)	Survivors (*n* = 296)	Non-Survivors (*n* = 88)	*p* Value
Age, years	64.7 (55.4–74.6)	63.3 (53.8–72.8)	70.9 (59.5–82.8)	<0.001
Male sex, *n* (%)	242 (63.0)	183 (61.8)	59 (67.0)	0.447
Diabetes mellitus, *n* (%)	119 (31.0)	84 (28.4)	35 (39.8)	0.058
APACHE II score	18.0 (14.0–22.0)	17.0 (12.8–20.0)	23.0 (20.0–26.0)	<0.001
Glasgow Coma Scale score	11.0 (10.0–13.0)	12.0 (10.0–14.0)	10.0 (8.0–11.0)	<0.001
Albumin, g/dL	2.1 (1.7–2.4)	2.2 (1.9–2.6)	1.7 (1.4–2.1)	<0.001
Lactate, mmol/L	2.0 (1.4–2.9)	1.7 (1.2–2.4)	2.9 (2.2–4.3)	<0.001
Lactate-to-albumin ratio	0.96 (0.57–1.60)	0.80 (0.52–1.25)	1.79 (1.20–2.69)	<0.001
Glucose variability (CV, %)	11.5 (7.5–16.6)	10.9 (6.8–16.0)	15.5 (10.8–20.0)	<0.001
**Primary diagnosis, *n* (%)**				<0.001
Sepsis	109 (28.4)	83 (28.0)	26 (29.5)	
Septic shock	54 (14.1)	28 (9.5)	26 (29.5)	
Pneumonia	105 (27.3)	84 (28.4)	21 (23.9)	
Trauma	38 (9.9)	32 (10.8)	6 (6.8)	
Postoperative	33 (8.6)	29 (9.8)	4 (4.5)	
Other	45 (11.7)	40 (13.5)	5 (5.7)	

Data are presented as medians (interquartile ranges) or numbers (%), as appropriate. Continuous variables were compared between survivors and non-survivors using the Mann–Whitney U test. Categorical variables were compared using Pearson’s χ^2^ test or Fisher’s exact test, as appropriate. The *p* value for primary diagnosis represents the overall comparison of the diagnostic distribution between groups. APACHE II, Acute Physiology and Chronic Health Evaluation II; CV, coefficient of variation.

**Table 2:** Univariable and primary multivariable Firth logistic regression analyses for 7-day post-landmark mortality.

Predictor	Univariable OR (95% CI)	*p* Value	Adjusted OR (95% CI)	*p* Value
Age, per 1-year increase	1.03 (1.02–1.05)	<0.001	—	—
APACHE II score, per 1-point increase	1.26 (1.19–1.34)	<0.001	1.19 (1.12–1.27)	<0.001
GCS score, per 1-point increase	0.54 (0.46–0.63)	<0.001	—	—
Albumin, per 1-g/dL increase	0.15 (0.08–0.26)	<0.001	—	—
Lactate, per 1-mmol/L increase	1.90 (1.57–2.31)	<0.001	—	—
LAR, per 1-unit increase	2.91 (2.17–3.90)	<0.001	1.97 (1.48–2.69)	<0.001
Glucose variability (CV), per 1-percentage-point increase	1.09 (1.06–1.13)	<0.001	1.07 (1.02–1.11)	0.002

Odds ratios and 95% confidence intervals were estimated using Firth penalized logistic regression. The primary multivariable model included APACHE II score, lactate-to-albumin ratio, and glucose variability. Univariable analyses were descriptive and were not used as a statistical screening procedure for predictor selection. Age and GCS were not entered separately into the primary multivariable model because they contribute to the APACHE II score. Albumin and lactate were not entered separately because they are the components of LAR. All continuous predictors were modeled without categorization. OR, odds ratio; CI, confidence interval; APACHE II, Acute Physiology and Chronic Health Evaluation II; GCS, Glasgow Coma Scale; LAR, lactate-to-albumin ratio; CV, coefficient of variation.

**Table 3:** Sequential incremental prognostic performance and bootstrap-corrected validation of APACHE II, LAR, and glucose variability.

Model	Predictors	AUC (95% CI)	ΔAUC vs. Previous Model (95% CI)	DeLong *p* Value	Apparent Brier Score	Optimism- Corrected C-Index	Optimism- Corrected Brier Score
M0	APACHE II	0.804 (0.755–0.854)	—	—	0.137	0.803	0.138
M1	APACHE II + LAR	0.844 (0.802–0.886)	+0.040 (0.014–0.065)	0.002	0.126	0.840	0.129
M2	APACHE II + LAR + GV	0.857 (0.816–0.898)	+0.013 (−0.004–0.030)	0.145	0.121	0.851	0.124

AUC differences were evaluated using paired DeLong tests. Internal validation was performed using 1000 nonparametric bootstrap resamples with model refitting. Optimism-corrected estimates were obtained by subtracting mean bootstrap optimism from the corresponding apparent performance measures. The overall increase in AUC from APACHE II alone to the final integrated model was 0.053 (95% CI: 0.022–0.083; *p* < 0.001). In conventional nested logistic-regression sensitivity analyses, addition of LAR improved model fit (likelihood-ratio χ^2^ = 25.6, df = 1; *p* < 0.001), and addition of GV after LAR also improved model fit (χ^2^ = 9.93, df = 1; *p* = 0.002). For the final integrated model, the optimism-corrected calibration intercept was −0.012 and the calibration slope was 0.985. APACHE II, Acute Physiology and Chronic Health Evaluation II; LAR, lactate-to-albumin ratio; GV, glucose variability; AUC, area under the receiver operating characteristic curve.

**Table 4:** Sensitivity analyses evaluating mean glycemia, temporal comparability, LAR decomposition, and nonlinear modeling of glucose variability. (**A**) Mean-glycemia-adjusted and temporally matched GV analyses. (**B**) LAR versus separate lactate and albumin terms. (**C**) Linear versus restricted cubic spline specification of GV.

(**A**)									
**Analysis**	**Reference model AUC (95% CI)**	**Extended** **model AUC** **(95% CI)**	**ΔAUC** **(95% CI)**	**DeLong *p***	**GV OR** **(95% CI)**	**GV *p***	**Corrected** **C-index,** **reference → extended**		**Corrected Brier,** **reference → extended**
0–48 h mean-glycemia adjusted	0.847 (0.806–0.888)	0.857 (0.817–0.897)	+0.010 (−0.008 to 0.027)	0.275	1.06 (1.02–1.11)	0.002	0.841 → 0.849		0.131 → 0.126
Temporally matched 0–24 h	0.848 (0.807–0.889)	0.852 (0.811–0.892)	+0.003 (−0.012 to 0.019)	0.651	1.05 (1.01–1.09)	0.008	0.841 → 0.843		0.131 → 0.127
(**B**)									
**Comparison**		**LAR model** **AUC (95% CI)**	**Separate-** **component model AUC (95% CI)**	ΔAUC * **(95% CI)**		***p*** **value**	**Brier,** **LAR model**		**Brier, separate** **components**
APACHE II + LAR vs. APACHE II + lactate + albumin		0.844 (0.802–0.886)	0.853 (0.812–0.893)	+0.009 (−0.004 to 0.021)		0.186	0.126		0.123
APACHE II + LAR + GV vs. APACHE II + lactate + albumin + GV		0.857 (0.816–0.898)	0.865 (0.825–0.904)	+0.007 (−0.006 to 0.021)		0.272	0.121		0.117
(**C**)									
**Model**	**Apparent AUC (95% CI)**		**Apparent Brier**	**Optimism-corrected C-index**		**Optimism-corrected Brier**		**Optimism-corrected calibration slope**	
Linear GV	0.857 (0.816–0.898)		0.121	0.852		0.124		0.990	
RCS GV	0.864 (0.824–0.904)		0.118	0.854		0.123		0.970	

(**A**) Reference/extended models: 0–48 h: APACHE II + LAR + mean glucose48 → + GV48, 0–24 h: APACHE II + LAR + mean glucose24 → + GV24. (**B**) * ΔAUC = separate-component model minus LAR model. (**C**) Additional RCS comparison: ΔAUC = +0.007 (95% bootstrap CI: −0.010 to 0.023; *p* = 0.396). Overall GV association: likelihood-ratio χ^2^ = 16.9, df = 3, *p* < 0.001. Evidence of nonlinearity: χ^2^ = 7.01, df = 2, *p* = 0.030. GV odds ratios are expressed per 1-percentage-point increase in glucose coefficient of variation. Mean glucose was modeled per 10 mg/dL increase. AUC differences were evaluated using paired DeLong tests except for the linear-versus-RCS comparison, for which the AUC difference was evaluated using 5000 paired bootstrap resamples. Optimism-corrected performance estimates were obtained using 1000 nonparametric bootstrap resamples with model refitting. The 24 h sensitivity analysis retained the same 48 h landmark cohort but restricted predictor information to the first 24 h. RCS modeling used four knots at the 5th, 35th, 65th, and 95th percentiles of GV. APACHE II, Acute Physiology and Chronic Health Evaluation II; LAR, lactate-to-albumin ratio; GV, glucose variability; CV, coefficient of variation; RCS, restricted cubic spline; AUC, area under the receiver operating characteristic curve; CI, confidence interval.

## Data Availability

The patient-level dataset analyzed in this study is not publicly available because it contains clinical information subject to patient confidentiality, institutional data-sharing policies, and applicable data-protection requirements. De-identified data may be made available from the corresponding author upon reasonable request, subject to institutional approval and applicable ethical and data-protection requirements. The complete R analysis scripts used for data preprocessing, derivation of glucose variability metrics, prognostic modeling, mean-glycemia-adjusted and temporally matched sensitivity analyses, lactate-to-albumin ratio decomposition, bootstrap internal validation, restricted cubic spline analyses, calibration assessment, and decision-curve analysis are publicly available in Zenodo (record 22142327) [18]. The repository also includes a data dictionary and supporting reproducibility documentation. Patient-level clinical data are not included in the public repository because of confidentiality, institutional data-sharing policies, and applicable data-protection requirements; access to de-identified data is governed by the Data Availability Statement.

## Supplementary Materials

The following supporting information can be downloaded at https://www.mdpi.com/article/10.3390/jcm15176925/s1: Figure S1: Decision-curve analysis of the sequential prognostic models; Table S1: Key Firth penalized logistic regression coefficients from mean-glycemia-adjusted, temporally matched, and LAR-component sensitivity analyses; Table S2: Apparent and optimism-corrected performance metrics from 1000-resample bootstrap internal validation; Table S3: Threshold-specific net-benefit estimates from decision-curve analyses of the sequential primary and sensitivity models; Table S4: Standardized absolute mortality risks and individual prediction differences for linear and restricted cubic spline glucose-variability models; Table S5: Exploratory ROC-derived cut-offs in the development cohort; Table S6: Clarifications and differences between the original ethics-approved protocol and the final analytical implementation; Supplementary File S1: Original ethics-approved study protocol.
