# Supplementary material for: Incremental Prognostic Value of Glucose Variability and the Lactate-to-Albumin Ratio Beyond APACHE II After a 48-Hour Landmark in Critically Ill Adults: A Retrospective Cohort Study
Source: J Clin Med. 2026 Sep 7;15(17):6925. doi: 10.3390/jcm15176925 (PMC13566276; doi:10.3390/jcm15176925)
Supplement: Supplementary file 1 [file jcm-15-06925-s001.zip › jcm-4532031-Supplementary File S1-Original ethics-approved study protocol..pdf]

Araştırmanın Adı: Yoğun Bakım Hastalarında Glukoz Variabilitesi ve Albümin/Laktat Oranı ile Erken Mortalite İlişkisinin Değerlendirilmesi: Retrospektif Gözlemsel Bir Çalışma.

Sorumlu Araştırmacı: (Öğretim üyesi, uzman vb.)

Adı / Soyadı	Unvanı	Görevi	İmzası	Yazışma Adresi
Oğuz Kağan BULUT	Uz. Dr.	Anestezi Uzm.		Elazığ Fethi Sekin Şehir Hast

Telefon: xxx E-Mail: oguzkaganbulut@hotmail.com

Diğer Araştırmacılar (Katkı sırasıyla)

Adı / Soyadı	Unvanı	Görevi	İmzası	Adresi
Sait Fatih ÖNER	Uz. Dr.	Anestezi Uzm.		Elazığ Fethi Sekin Şehir Hast

Telefon: xxx E-Mail: sfatihoner@gmail.com

ARAŞTIRMANIN NİTELİĞİ

- ☒ Münferit Araştırma
- ☐ Doktora Tezi
- ☐ Uzmanlık Tezi
- ☐ Yüksek Lisans Tezi
- ☐ TÜBİTAK, BAP Projesi vb.
- ☐ Diğer (Türünü belirtiniz):

ARAŞTIRMANIN ÖNGÖRÜLEN SÜRESİ:

Planlanan Yaklaşık Süre Tarihi *(Etik kurulu onay tarihinden sonraki bir tarih olmalı)*: **Etik kurul onayı alındıktan 3 ay sonra**

Planlanan Bitiş Tarihi: 20.06.2026

ARAŞTIRMANIN GEREÇ VE YÖNTEMİ

Araştırmanın örneklemini oluşturan istatiksel yöntem(ler birey sayısı, araştırmanın hipotezleri, araştırmaya alma ve dışlama kriterleri, veri toplama araç ve yöntemleri, kullanılacak) detaylı ve anlaşılır yazılmalıdır.

Çalışmaya, Elazığ Fethi Sekin Şehir Hastanesi Anesteziyoloji ve Reanimasyon Yoğun Bakım Ünitesi’nde 01.01.2023–31.12.2025 tarihleri arasında takip edilen hastalar dahil edilecektir. Hastane bilgi yönetim sistemi ve yoğun bakım dijital kayıtları geriye dönük olarak taranacaktır.

Glukoz variabilitesi; ilk 48 saat içindeki en az 6 glukoz ölçümüne dayanarak standart sapma, varyasyon katsayısı ve MAGE (Mean Amplitude of Glycemic Excursions) yöntemleri ile hesaplanacaktır. Albümin/laktat oranı, yoğun bakıma kabulden sonraki ilk 24 saat içinde ölçülen en erken albümin ve laktat değerleri kullanılarak hesaplanacaktır.

Erken mortalite, yoğun bakım yatışının ilk 7 günü içinde gerçekleşen ölüm olarak tanımlanacaktır. Tüm veriler anonim hale getirilecek, kimlik bilgileri kayıt dışı bırakılacaktır.

H0: Yoğun bakım hastalarında glukoz variabilitesi ve albümin/laktat oranı ile erken mortalite arasında anlamlı bir ilişki yoktur.

H1: Yoğun bakım hastalarında glukoz variabilitesi ve albümin/laktat oranı ile erken mortalite arasında anlamlı bir ilişki vardır.

Araştırmanın bütçesi: Sorumlu araştırmacı tarafından karşılanacaktır.

Araştırmaya dahil edilme kriterleri:

- ≥18 yaş hastalar
- Belirtilen tarih aralığında yoğun bakımda izlenmiş olmak
- Yoğun bakımda en az 48 saat takip edilmiş olmak
- İlk 48 saat içinde en az 6 adet kan glukoz ölçümü bulunması
- İlk 24 saat içinde albümin ve laktat ölçümü bulunması
- Klinik ve laboratuvar verilerine eksiksiz olarak ulaşılabilmesi

Araştırmaya dahil edilmeme kriterleri:

- 18 yaş altı hastalar
- Yoğun bakımda 48 saatten kısa süre izlenen hastalar
- Yeterli glukoz ölçümü olmayan hastalar
- Albümin veya laktat değeri eksik olan hastalar
- Veri kayıtları eksik veya tutarsız olan olgular
- Aynı yatış dönemine ait mükerrer kayıtlar

Veri toplama aracı/araçları:
Veriler hastane bilgi yönetim sistemi, yoğun bakım hasta izlem formları ve laboratuvar veri tabanı üzerinden elde edilecektir. Toplanacak değişkenler şunlardır:

- Demografik veriler
- Yatış tanısı ve komorbiditeler
- APACHE II skoru
- Seri kan glukoz ölçümleri
- Serum albümin düzeyi
- Serum laktat düzeyi
- Hesaplanan glukoz variabilite parametreleri
- Albümin/laktat oranı
- Yoğun bakım kalış süresi
- İlk 7 gün mortalite durumu

Veriler Excel/SPSS veri tabanına kodlanmış hasta numarası ile aktarılacaktır.

Kullanılacak istatiksel yöntem(ler)

Elde edilen veriler istatistik paket programı kullanılarak analiz edilecektir. Sürekli değişkenlerin dağılımı normallik testleri ile değerlendirilecek, normal dağılıma uyan veriler ortalama±standart sapma, uymayanlar medyan (minimum–maksimum) olarak sunulacaktır.

Kategorik değişkenler sayı ve yüzde ile ifade edilecektir. Yaşayan ve erken mortalite gelişen hasta grupları arasında sürekli değişkenler uygunluğa göre parametrik veya non-parametrik testlerle karşılaştırılacaktır. Kategorik değişkenler ki-kare veya Fisher exact test ile analiz edilecektir. Glukoz variabilitesi parametreleri ve albümin/laktat oranının erken mortalite üzerindeki etkisini değerlendirmek amacıyla çok değişkenli lojistik regresyon modeli kurulacaktır. Bağımsız değişkenler klinik anlamlılık ve tek değişkenli analiz sonuçlarına göre modele dahil edilecektir. Ayırt edici gücü değerlendirmek için ROC eğrisi analizi yapılacaktır. İstatistiksel anlamlılık düzeyi $p<0.05$ olarak kabul edilecektir.

ARAŞTIRMANIN KAPSAMI

☐ Gözlemsel araştırma
☒ Dosya ve görüntü kayıtları gibi retrospektif arşiv taraması
☐ Kan, idrar, doku, görüntü gibi biyokimya, mikrobiyoloji, patoloji ve radyoloji koleksiyon materyalleriyle veya rutin muayene, tetkik, tahlil ve tedavi işlemleri sırasında elde edilmiş materyalle yapılacak araştırma
☐ Hücre veya doku kültürü araştırması
☐ Gen tedavisi klinik araştırmaları dışında kalan ve tanımlamaya yönelik genetik materyalle yapılacak araştırma
☐ Hemşirelik faaliyetlerinin sınırları içerisinde yapılacak araştırma
☐ Bilgisayar ortamında test, mülakat, ses veya görüntü kaydı ile toplanacak verilerin kullanılacağı araştırma
☐ Biyomedikal ve/veya ekoloji ile ilgili araştırma
☐ Sağlık eğitimi faaliyetleri ile ilgili araştırma
☐ Antropometrik ölçümlere dayalı araştırma
☐ Egzersiz gibi vücut fizyolojisi ile ilgili araştırma
☐ Beslenme/diyet ile ilgili araştırma
☐ Yaşam alışkanlıklarının değerlendirilmesi ile ilgili araştırma
☐ Vücut dışında kullanılan tıbbi tanı cihazları ile yapılan performans değerlendirmesi ile ilgili araştırma
☐ Anket araştırması
☐ Diğer (kısaca açıklayınız):

ARAŞTIRMANIN AMACI / GEREKÇESİ:

Araştırmanın gerekçesi vancoover sistemine ve literatüre dayandırılmalı referanslar verilmeli

Yoğun bakım ünitelerinde izlenen hastalarda mortalite oranları yüksek seyretmekte olup, erken dönemde kötü prognozu öngörebilecek güvenilir, kolay ulaşılabilir ve klinik pratiğe entegre edilebilir biyobelirteçlerin belirlenmesi kritik öneme sahiptir. Son yıllarda yalnızca ortalama kan glukoz düzeyinin değil, glukoz düzeylerindeki dalgalanmanın derecesini yansıtan **glukoz variabilitesinin** de klinik sonuçlar üzerinde belirleyici rol oynadığı gösterilmiştir. Artmış glukoz variabilitesinin oksidatif stres, endotelyal disfonksiyon ve inflamatuvar yanıt artışı ile ilişkili olduğu; bu mekanizmalar üzerinden organ yetmezliği ve mortalite riskini artırabileceği bildirilmektedir (1,2).

Kritik hastalarda yapılan güncel çalışmalarda, glisemik oynaklığın mortalite, yoğun bakım kalış süresi ve komplikasyon oranları ile bağımsız ilişkili olabileceği; hatta bazı hasta gruplarında ortalama glukoz düzeyinden daha güçlü bir prognostik gösterge olduğu vurgulanmıştır (2,3). Bu nedenle glukoz variabilitesi, yoğun bakım pratiğinde giderek daha fazla dikkate alınan metabolik risk göstergelerinden biri haline gelmiştir.

Laktat düzeyi ise doku hipoperfüzyonunun ve hücresel hipoksinin yerleşik bir biyokimyasal göstergesi olmakla birlikte, tek başına kullanıldığında prognostik gücü hasta popülasyonuna göre değişkenlik gösterebilmektedir. Son yıllarda yapılan çalışmalarda, laktat düzeyinin serum albümin ile oranlanmasıyla elde edilen **albümin/laktat oranının**, hem metabolik rezervi hem de perfüzyon durumunu birlikte yansıttığı ve tek başına laktata kıyasla mortaliteyi öngörmede daha güçlü bir parametre olabileceği gösterilmiştir. Özellikle sepsis ve kardiyak arrest sonrası kritik hastalarda albümin/laktat oranının düşük olmasının hastane içi mortalite ile anlamlı ve bağımsız ilişkili olduğu bildirilmiştir (4).

Buna karşın literatürde glukoz variabilitesi ile albümin/laktat oranının çoğunlukla ayrı ayrı incelendiği, bu iki metabolik ve perfüzyon temelli göstergenin birlikte değerlendirildiği çalışmaların ise sınırlı olduğu görülmektedir. Metabolik instabiliteyi yansıtan glukoz variabilitesi ile perfüzyon ve protein rezerv göstergesi olan albümin/laktat oranının kombine değerlendirilmesi, yoğun bakım hastalarında erken mortalite risk sınıflamasına katkı sağlayabilecek bütüncül bir yaklaşım sunabilir (5).

Bu çalışmanın amacı, yoğun bakım ünitesinde izlenen erişkin hastalarda glukoz variabilitesi ve albümin/laktat oranının erken mortalite ile ilişkisini retrospektif olarak değerlendirmek, bu parametrelerin bağımsız öngördürücü değerini ortaya koymak ve klinik risk değerlendirme modellerine katkı sağlayabilecek pratik biyobelirteçler olup olmadığını araştırmaktır.

ARAŞTIRMANIN YAPILACAĞI MERKEZ/MERKEZLER
(Araştırma verisi çevrimiçi toplanacaksa belirtilmelidir)

Elazığ Fethi Sekin Şehir Hastanesi

REFERANSLAR *(En fazla 5 referansve vancoover Word kullanılarak yazılmalıdır)*

1. Kim HJ et al. Albumin-to-lactate ratio as a predictor of in-hospital mortality in patients with sepsis. Am J Emerg Med, 2022.
2. Park J et al. Prognostic value of the albumin-to-lactate ratio in critically ill patients with cardiac arrest. Resuscitation, 2023.
3. Wernly B et al. Blood glucose variability and mortality in critical illness. Crit Care, 2020.
4. Krinsley JS, Preiser JC. Glycemic variability and outcomes in critically ill patients. Curr Opin Clin Nutr Metab Care, 2021.
5. Zampieri FG et al. Lactate-based metrics and outcomes in critical care. Intensive Care Med, 2021.
