# Supplementary material for: Incremental Prognostic Value of Glucose Variability and the Lactate-to-Albumin Ratio Beyond APACHE II After a 48-Hour Landmark in Critically Ill Adults: A Retrospective Cohort Study
Source: J Clin Med. 2026 Sep 7;15(17):6925. doi: 10.3390/jcm15176925 (PMC13566276; doi:10.3390/jcm15176925)
Supplement: Supplementary file 1 [file jcm-15-06925-s001.zip › jcm-4532031-supplementary.pdf]

Supplementary Materials

Supplementary Figure S1. Decision-curve analysis of the sequential prognostic models

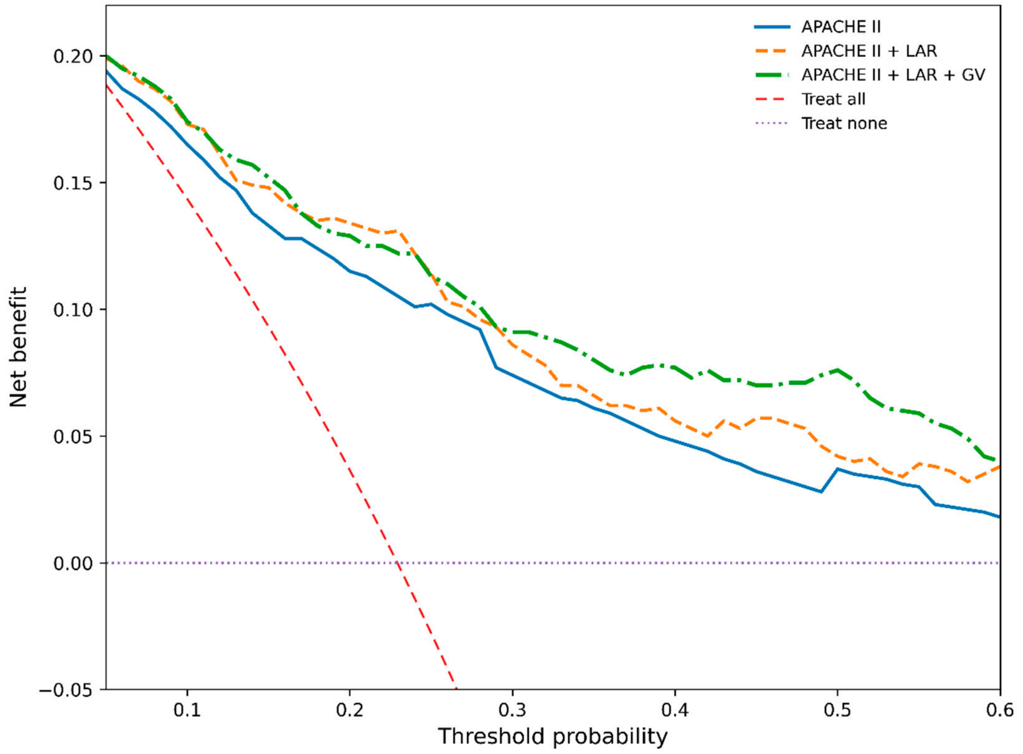

Net benefit is shown across threshold probabilities from 0.05 to 0.60 for APACHE II alone, APACHE II + LAR, and APACHE II + LAR + GV, together with treat-all and treat-none strategies. The integrated model showed greater net benefit than APACHE II alone across the evaluated range, whereas the incremental net benefit attributable specifically to GV after addition of LAR was smaller and threshold-dependent. The y-axis was truncated at −0.05 to facilitate visualization of differences between the prognostic models; the treat-all strategy falls below the displayed range at higher threshold probabilities. APACHE II, Acute Physiology and Chronic Health Evaluation II; LAR, lactate-to-albumin ratio; GV, glucose variability.

Supplementary Table S1. Key Firth penalized logistic regression coefficients from mean-glycemia-adjusted, temporally matched, and LAR-component sensitivity analyses

Sensitivity analysis	Predictor	OR (95% CI)	p value
0–48 h mean-glycemia-adjusted model without LAR	Mean glucose, per 10-mg/dL increase	1.08 (0.97–1.20)	0.148
	GV48, per 1-percentage-point increase in CV	1.07 (1.03–1.11)	<0.001
0–48 h mean-glycemia-adjusted model with LAR	LAR, per 1-unit increase	1.93 (1.44–2.65)	<0.001
	Mean glucose, per 10-mg/dL increase	1.04 (0.93–1.16)	0.539
	GV48, per 1-percentage-point increase in CV	1.06 (1.02–1.11)	0.002
Temporally matched 0–24 h model	LAR, per 1-unit increase	1.88 (1.41–2.57)	<0.001
	Mean glucose, per 10-mg/dL increase	1.07 (0.96–1.20)	0.220
	GV24, per 1-percentage-point increase in CV	1.05 (1.01–1.09)	0.008
LAR decomposition: APACHE II + lactate + albumin	Lactate, per 1-mmol/L increase	1.38 (1.14–1.68)	<0.001
	Albumin, per 1-g/dL increase	0.30 (0.16–0.57)	<0.001
LAR decomposition: APACHE II + lactate + albumin + GV48	Lactate, per 1-mmol/L increase	1.35 (1.11–1.64)	0.002
	Albumin, per 1-g/dL increase	0.28 (0.14–0.54)	<0.001
	GV48, per 1-percentage-point increase in CV	1.07 (1.03–1.12)	0.001

Odds ratios and 95% confidence intervals were estimated using Firth penalized logistic regression. The first 0–48-hour mean-glycemia-adjusted model included APACHE II, mean glucose calculated from the six glucose measurements obtained closest to 0, 8, 16, 24, 32, and 48 hours, and GV48. The second 0–48-hour model additionally included LAR. The temporally matched 0–24-hour model included APACHE II, LAR, mean glucose calculated from the four measurements obtained closest to 0, 8, 16, and 24 hours, and GV24. The LAR-component sensitivity analyses replaced LAR with lactate and albumin entered as separate terms. APACHE II coefficients are not repeated because the table is intended to display the biomarker and mean-glycemia coefficients directly relevant to the sensitivity analyses. GV, glucose variability; CV, coefficient of variation; LAR, lactate-to-albumin ratio; APACHE II, Acute Physiology and Chronic Health Evaluation II; OR, odds ratio; CI, confidence interval.

Supplementary Table S2. Apparent and optimism-corrected performance metrics from 1,000-resample bootstrap internal validation

Model	Apparent C-index	Optimism-corrected C-index	Optimism-corrected Somers' Dxy	Apparent Brier score	Optimism-corrected Brier score	Optimism-corrected calibration intercept	Optimism-corrected calibration slope
APACHE II	0.804	0.803	0.605	0.137	0.138	−0.008	1.010
APACHE II + LAR	0.844	0.840	0.680	0.126	0.129	−0.011	0.989
APACHE II + LAR + GV48	0.857	0.851	0.703	0.121	0.124	−0.012	0.985
APACHE II + LAR + mean glucose48	0.847	0.841	0.681	0.127	0.131	−0.015	0.976
APACHE II + LAR + mean glucose48 + GV48	0.857	0.849	0.697	0.121	0.126	−0.016	0.973
APACHE II + LAR + mean glucose24	0.848	0.841	0.683	0.126	0.131	−0.015	0.977
APACHE II + LAR + mean glucose24 + GV24	0.852	0.843	0.686	0.122	0.127	−0.015	0.970

Internal validation was performed using 1,000 nonparametric bootstrap resamples with complete model refitting in each resample. Optimism was estimated as the difference between model performance in each bootstrap sample and the performance of the bootstrap-fitted model when applied to the original development cohort. Optimism-corrected estimates were obtained by subtracting mean optimism from the corresponding apparent estimates. The 0–48-hour mean-glycemia-adjusted models used mean glucose derived from the same six glucose measurements used to calculate GV48. The temporally matched 0–24-hour models used mean glucose and GV calculated from the four measurements closest to 0, 8, 16, and 24 hours; these analyses retained the same 48-hour landmark cohort. APACHE II, Acute Physiology and Chronic Health Evaluation II; LAR, lactate-to-albumin ratio; GV, glucose variability; Dxy, Somers' rank correlation coefficient.

Supplementary Table S3. Threshold-specific net-benefit estimates from decision-curve analyses of the sequential primary and sensitivity models

Panel A. Sequential primary-model decision-curve analysis

Threshold	APACHE II	APACHE II + LAR	APACHE II + LAR + GV	Δ LAR vs APACHE	Δ GV vs APACHE + LAR	Δ Final vs APACHE
0.05	0.194	0.199	0.200	0.0054	0.0014	0.0067
0.06	0.187	0.196	0.195	0.0085	−0.0001	0.0084
0.07	0.183	0.190	0.192	0.0069	0.0026	0.0095
0.08	0.178	0.187	0.188	0.0088	0.0013	0.0101
0.09	0.172	0.182	0.183	0.0101	0.0007	0.0108
0.10	0.165	0.173	0.174	0.0084	0.0015	0.0098
0.11	0.159	0.171	0.170	0.0119	−0.0013	0.0106
0.12	0.152	0.161	0.163	0.0097	0.0018	0.0115
0.13	0.147	0.151	0.159	0.0049	0.0072	0.0121
0.14	0.138	0.149	0.157	0.0116	0.0078	0.0193
0.15	0.133	0.148	0.152	0.0153	0.0041	0.0195
0.16	0.128	0.142	0.147	0.0134	0.0056	0.0190
0.17	0.128	0.138	0.138	0.0095	0.0001	0.0096
0.18	0.124	0.135	0.133	0.0109	−0.0015	0.0094
0.19	0.120	0.136	0.130	0.0165	−0.0058	0.0107
0.20	0.115	0.134	0.129	0.0189	−0.0052	0.0137
0.21	0.113	0.132	0.125	0.0191	−0.0068	0.0123
0.22	0.109	0.130	0.125	0.0217	−0.0059	0.0158
0.23	0.105	0.131	0.122	0.0259	−0.0091	0.0168
0.24	0.101	0.122	0.122	0.0211	0.0001	0.0212
0.25	0.102	0.114	0.113	0.0122	−0.0009	0.0113
0.26	0.098	0.103	0.110	0.0047	0.0073	0.0120
0.27	0.095	0.101	0.105	0.0064	0.0041	0.0105
0.28	0.092	0.096	0.101	0.0048	0.0051	0.0098
0.29	0.077	0.093	0.093	0.0162	0.0001	0.0163
0.30	0.074	0.086	0.091	0.0123	0.0045	0.0167
0.31	0.071	0.082	0.091	0.0111	0.0085	0.0195
0.32	0.068	0.078	0.089	0.0100	0.0112	0.0211
0.33	0.065	0.070	0.087	0.0051	0.0168	0.0218
0.34	0.064	0.070	0.084	0.0064	0.0145	0.0209

0.35	0.061	0.066	0.080	0.0052	0.0134	0.0186
0.36	0.059	0.062	0.076	0.0029	0.0148	0.0177
0.37	0.056	0.062	0.074	0.0061	0.0119	0.0181
0.38	0.053	0.060	0.077	0.0070	0.0162	0.0232
0.39	0.050	0.061	0.078	0.0116	0.0164	0.0279
0.40	0.048	0.056	0.077	0.0087	0.0208	0.0295
0.41	0.046	0.053	0.073	0.0076	0.0192	0.0268
0.42	0.044	0.050	0.076	0.0067	0.0258	0.0324
0.43	0.041	0.056	0.072	0.0143	0.0163	0.0307
0.44	0.039	0.053	0.072	0.0140	0.0186	0.0326
0.45	0.036	0.057	0.070	0.0213	0.0125	0.0339
0.46	0.034	0.057	0.070	0.0231	0.0130	0.0361
0.47	0.032	0.055	0.071	0.0228	0.0156	0.0385
0.48	0.030	0.053	0.071	0.0230	0.0176	0.0407
0.49	0.028	0.046	0.074	0.0180	0.0281	0.0462
0.50	0.037	0.042	0.076	0.0052	0.0339	0.0391
0.51	0.035	0.040	0.072	0.0046	0.0319	0.0365
0.52	0.034	0.041	0.065	0.0069	0.0243	0.0312
0.53	0.033	0.036	0.061	0.0039	0.0248	0.0286
0.54	0.031	0.034	0.060	0.0034	0.0252	0.0286
0.55	0.030	0.039	0.059	0.0093	0.0200	0.0292
0.56	0.023	0.038	0.055	0.0147	0.0170	0.0317
0.57	0.022	0.036	0.053	0.0140	0.0173	0.0313
0.58	0.021	0.032	0.049	0.0117	0.0166	0.0283
0.59	0.020	0.035	0.042	0.0159	0.0067	0.0226
0.60	0.018	0.038	0.040	0.0195	0.0026	0.0221

Panel B. Mean-glycemia-adjusted and temporally matched sensitivity decision curves

Threshold	48-h reference	48-h + GV	ΔGV48	24-h reference	24-h + GV	ΔGV24
0.05	0.199	0.201	0.0015	0.200	0.200	0.0007
0.06	0.196	0.198	0.0027	0.196	0.196	−0.0003
0.07	0.189	0.194	0.0043	0.192	0.192	0.0001
0.08	0.187	0.188	0.0010	0.185	0.190	0.0044
0.09	0.183	0.186	0.0031	0.184	0.186	0.0026

0.10	0.175	0.177	0.0023	0.182	0.182	0.0000
0.11	0.173	0.165	−0.0085	0.173	0.179	0.0052
0.12	0.165	0.164	−0.0012	0.167	0.170	0.0030
0.13	0.156	0.158	0.0016	0.164	0.157	−0.0070
0.14	0.151	0.157	0.0056	0.155	0.153	−0.0013
0.15	0.150	0.152	0.0018	0.148	0.146	−0.0021
0.16	0.141	0.149	0.0082	0.140	0.144	0.0036
0.17	0.136	0.139	0.0027	0.139	0.139	0.0005
0.18	0.134	0.135	0.0014	0.134	0.135	0.0017
0.19	0.133	0.130	−0.0034	0.132	0.134	0.0020
0.20	0.131	0.128	−0.0033	0.128	0.125	−0.0033
0.21	0.132	0.127	−0.0054	0.130	0.122	−0.0081
0.22	0.132	0.125	−0.0063	0.128	0.119	−0.0089
0.23	0.126	0.125	−0.0005	0.127	0.117	−0.0099
0.24	0.117	0.122	0.0045	0.116	0.117	0.0011
0.25	0.111	0.113	0.0017	0.106	0.106	0.0000
0.26	0.103	0.108	0.0046	0.103	0.100	−0.0035
0.27	0.100	0.104	0.0032	0.099	0.095	−0.0036
0.28	0.098	0.098	0.0003	0.095	0.091	−0.0036
0.29	0.093	0.094	0.0012	0.092	0.090	−0.0015
0.30	0.088	0.091	0.0030	0.087	0.094	0.0071
0.31	0.083	0.089	0.0059	0.087	0.086	−0.0014
0.32	0.079	0.084	0.0051	0.084	0.087	0.0025
0.33	0.073	0.083	0.0103	0.079	0.080	0.0013
0.34	0.069	0.084	0.0159	0.075	0.074	−0.0012
0.35	0.067	0.082	0.0150	0.069	0.076	0.0072
0.36	0.065	0.079	0.0140	0.066	0.075	0.0088
0.37	0.068	0.081	0.0129	0.068	0.071	0.0024
0.38	0.070	0.079	0.0094	0.067	0.070	0.0038
0.39	0.060	0.076	0.0164	0.067	0.067	0.0007
0.40	0.054	0.073	0.0191	0.063	0.066	0.0035
0.41	0.057	0.078	0.0211	0.058	0.068	0.0098
0.42	0.054	0.070	0.0161	0.051	0.066	0.0146
0.43	0.051	0.068	0.0169	0.051	0.068	0.0176
0.44	0.048	0.070	0.0212	0.045	0.064	0.0186

0.45	0.052	0.068	0.0156	0.047	0.062	0.0147
0.46	0.052	0.077	0.0245	0.048	0.060	0.0126
0.47	0.053	0.073	0.0202	0.045	0.063	0.0176
0.48	0.045	0.074	0.0282	0.048	0.066	0.0180
0.49	0.044	0.077	0.0334	0.044	0.069	0.0257
0.50	0.042	0.076	0.0339	0.042	0.068	0.0260
0.51	0.043	0.064	0.0213	0.043	0.069	0.0265
0.52	0.046	0.060	0.0135	0.043	0.060	0.0163
0.53	0.042	0.058	0.0163	0.042	0.053	0.0111
0.54	0.038	0.054	0.0165	0.035	0.052	0.0170
0.55	0.036	0.059	0.0231	0.036	0.048	0.0116
0.56	0.028	0.055	0.0263	0.035	0.047	0.0118
0.57	0.030	0.048	0.0182	0.034	0.049	0.0147
0.58	0.030	0.046	0.0166	0.036	0.051	0.0150
0.59	0.035	0.042	0.0067	0.038	0.050	0.0116
0.60	0.035	0.044	0.0091	0.034	0.048	0.0143

Net benefit was evaluated at threshold probabilities from 0.05 to 0.60 in increments of 0.01. In Panel A, ΔLAR vs APACHE represents the net-benefit difference between APACHE II + LAR and APACHE II alone; ΔGV vs APACHE + LAR represents the difference after adding GV to APACHE II + LAR; and ΔFinal vs APACHE represents the difference between the final integrated model and APACHE II alone. In Panel B, the 48-hour reference model included APACHE II, LAR, and mean glucose48, whereas the extended model additionally included GV48. The 24-hour reference model included APACHE II, LAR, and mean glucose24, whereas the extended model additionally included GV24. Positive differences favor the extended model; negative differences favor the corresponding reference model. DCA, decision-curve analysis; APACHE II, Acute Physiology and Chronic Health Evaluation II; LAR, lactate-to-albumin ratio; GV, glucose variability.

Supplementary Table S4. Standardized absolute mortality risks and individual prediction differences for linear and restricted cubic spline glucose-variability models

Panel A. Standardized predicted mortality risks at selected GV percentiles

GV percentile	GV (CV, %)	Linear-model standardized risk, %	RCS-model standardized risk, %	RCS − linear difference, percentage points
5th	3.58	15.4	6.2	−9.25
25th	7.53	17.9	19.0	+1.07
50th	11.5	20.8	26.3	+5.56
75th	16.6	24.9	22.1	−2.79
90th	21.5	29.2	25.0	−4.21
95th	24.8	32.4	30.0	−2.41

Panel B. Individual prediction differences between linear and RCS models

**Measure**	**Result**
Patients evaluated, n	384
Median absolute difference in predicted mortality risk	2.8 percentage points
Patients with absolute difference >5 percentage points	26.0%
Patients with absolute difference >10 percentage points	4.7%

Standardized mortality risks were estimated by assigning the same GV value to all patients at each selected percentile while retaining each patient's observed APACHE II and LAR values, and then averaging the resulting predicted probabilities. The linear and RCS models otherwise used the same APACHE II and LAR adjustment set. The RCS model used four knots located at the 5th, 35th, 65th, and 95th percentiles of the observed GV distribution. Differences in Panel A are signed as RCS minus linear and are expressed in percentage points. Individual prediction differences in Panel B are absolute differences between the two model-derived probabilities at each patient's observed GV value. GV, glucose variability; CV, coefficient of variation; RCS, restricted cubic spline; APACHE II, Acute Physiology and Chronic Health Evaluation II; LAR, lactate-to-albumin ratio.

Supplementary Table S5. Exploratory ROC-derived cut-offs in the development cohort

Predictor / model	Exploratory cut-off	Sensitivity, %	Specificity, %
APACHE II score	21.0	70.5	76.7
Lactate-to-albumin ratio	1.32	73.9	78.7
Glucose variability (CV)	9.75%	85.2	44.6
Integrated APACHE II + LAR + GV model predicted probability	0.240	76.1	79.4

Cut-offs were derived by maximizing the Youden index within the same development cohort used for model estimation. These thresholds are exploratory and have not been externally validated; therefore, they should not be interpreted as recommended clinical decision thresholds. For the integrated model, the reported cut-off refers to the predicted probability of 7-day post-landmark mortality. APACHE II, Acute Physiology and Chronic Health Evaluation II; LAR, lactate-to-albumin ratio; GV, glucose variability; CV, coefficient of variation.

Supplementary Table S6. Clarifications and differences between the original ethics-approved protocol and the final analytical implementation

Protocol item	Original protocol	Final implementation	Rationale
GV metrics	SD, CV, MAGE	CV primary; SD calculated; MAGE not calculated	Six-point retrospective sampling was insufficient for reliable MAGE
Ratio terminology	Albumin/lactate	Lactate/albumin (LAR)	Nomenclature error in original ethics form; final analysis consistently used lactate divided by albumin
Mortality window	First 7 ICU days	7 days after 48-h landmark	Ensures predictor ascertainment precedes outcome and defines a coherent conditional landmark estimand
Predictor selection	Candidate predictors defined on clinical grounds; univariable associations with p<0.10 considered supportive evidence	APACHE II, LAR and GV clinically defined; univariable analyses descriptive	Avoided data-driven p-value screening in final prognostic model

SD, standard deviation; CV, coefficient of variation; MAGE, mean amplitude of glycemic excursions; LAR, lactate-to-albumin ratio; ICU, intensive care unit; APACHE II, Acute Physiology and Chronic Health Evaluation II.
